# The Diversity of *Escherichia coli* Pathotypes and Vaccination Strategies against This Versatile Bacterial Pathogen

**DOI:** 10.3390/microorganisms11020344

**Published:** 2023-01-30

**Authors:** Pravil Pokharel, Sabin Dhakal, Charles M. Dozois

**Affiliations:** 1 Centre Armand-Frappier Santé Biotechnologie, Institut National de la Recherche Scientifique (INRS), 531 Boul des Prairies, Laval, QC H7V 1B7, Canada; 2 Centre de Recherche en Infectiologie Porcine et Avicole (CRIPA), Faculté de Médecine Vétérinaire, Université de Montréal Saint-Hyacinthe, Saint-Hyacinthe, QC J2S 2M2, Canada; 3 Pasteur Network, Laval, QC H7V 1B7, Canada

**Keywords:** *Escherichia coli*, bacterial infection, antimicrobial resistance, diarrhea, colibacillosis, urinary tract infection, sepsis, poultry, antimicrobial therapy, animal health

## Abstract

*Escherichia coli* (*E. coli*) is a gram-negative bacillus and resident of the normal intestinal microbiota. However, some *E. coli* strains can cause diseases in humans, other mammals and birds ranging from intestinal infections, for example, diarrhea and dysentery, to extraintestinal infections, such as urinary tract infections, respiratory tract infections, meningitis, and sepsis. In terms of morbidity and mortality, pathogenic *E. coli* has a great impact on public health, with an economic cost of several billion dollars annually worldwide. Antibiotics are not usually used as first-line treatment for diarrheal illness caused by *E. coli* and in the case of bloody diarrhea, antibiotics are avoided due to the increased risk of hemolytic uremic syndrome. On the other hand, extraintestinal infections are treated with various antibiotics depending on the site of infection and susceptibility testing. Several alarming papers concerning the rising antibiotic resistance rates in *E. coli* strains have been published. The silent pandemic of multidrug-resistant bacteria including pathogenic *E. coli* that have become more difficult to treat favored prophylactic approaches such as *E. coli* vaccines. This review provides an overview of the pathogenesis of different pathotypes of *E. coli*, the virulence factors involved and updates on the major aspects of vaccine development against different *E. coli* pathotypes.

## 1. Introduction

Vaccines are a major asset for the reduction of the burden of infectious diseases worldwide. They can provide long-term immunity, cheaper modalities than diagnosis and treatment of the infections after they have started, and most importantly—can prevent diseases from occurring in susceptible populations or animal species. Despite the potential benefits of vaccines, we do not see much enthusiasm for the development of vaccines against *E. coli*, particularly for human health. There could be scientific, financial, legal, or political barriers, although increased antimicrobial resistance may promote vaccine development if such pathogens become difficult to treat. Herein, we have summarized aspects of each pathotype of *E. coli* before presenting vaccine strategies, since different antigens or components (inactivated whole cells, O antigen, fimbriae, adhesins, enterotoxins, outer membrane proteins (OMPs)) have been used against different *E. coli* pathotypes. Some of the vaccine strategies have been licensed and shown to be efficacious, but most have only been at the developmental stage, as detailed below.

### 1.1. Escherichia coli

*Escherichia coli* (*E. coli*) is one of the most intensively studied model organisms in microbiology and molecular biology research [1,2]. *E. coli* is a well-known commensal bacterium that is among the first colonizing bacteria in the gut after birth. However, in immunosuppressed patients or in healthy individuals whose physical, anatomical and physiological barriers have been compromised, *E. coli* can cause severe systemic infections [3,4]. Further, due to the genetic variability some *E. coli* strains are different from their commensal counterparts and encode specific virulence traits that render them capable of causing disease in a variety of animals. Pathogenic *E. coli* are broadly divided into two groups, extraintestinal pathogenic *E. coli* (ExPEC) and intestinal pathogenic *E. coli* (InPEC) [3,5,6,7]. Depending on the presence of specific virulence factors, mechanisms of infection, tissue tropism, interactions with host cells and clinical symptoms, *E. coli* can be categorized into various pathotypes. These include: (i) Enteropathogenic *E. coli* (EPEC), a cause of acute and prolonged diarrhea in infants; (ii) Enterohemorrhagic *E. coli* (EHEC), which can cause hemorrhagic colitis and hemolytic uremic syndrome (HUS); (iii) Enterotoxigenic *E. coli* (ETEC), a major cause of travelers’ diarrhea; (iv) Enteroaggregative *E. coli* (EAEC), a cause of acute and chronic diarrhea; (v) Diffusely adherent *E. coli* (DAEC) which is associated with watery diarrhea in young children; (vi) Enteroinvasive *E. coli* (EIEC), a cause of dysentery and watery diarrhea; (vii) Adherent-Invasive *E. coli* (AIEC) which has been associated in the pathogenesis of inflammatory bowel disease (IBD); (viii) Uropathogenic *E. coli* (UPEC), a common cause of urinary tract infections (UTI); (ix) Neonatal meningitis *E. coli* (NMEC), a top cause of neonatal meningitis; (x) Septicemia-associated *E.* coli (SEPEC), which can cause bacteremia and sepsis; (xi) Avian pathogenic *E. coli* (APEC), which can cause severe respiratory and systemic infections in poultry. *E. coli* can also be classified into distinct phylogenetic lineages: A, B1, B2, D1, D2, E and clade I. Group A mostly represents non-pathogenic *E. coli* that reside along the gastrointestinal tract mucosa. Phylogroup B1 contains both commensal and some strains belonging to the EHEC pathotype. D1, D2, and E represent InPEC. Many ExPEC belongs to group B2. *E. coli* strains that are genetically diverse but phenotypically indistinguishable are grouped into cryptic clade I [5,8,9,10]. Studies indicate that some APEC and ExPEC strains are phylogenetically closely related and share certain virulence genes [11]. APEC and other avian *E. coli* may cause a wide variety of intestinal and extraintestinal infections [12,13,14,15], and in some cases, *E. coli* from poultry may be a reservoir of human ExPEC and InPEC isolates [16,17].

*E. coli* is one of the most genetically versatile microorganisms and can colonize and persist in primary (bird/animal/human host-associated) and secondary (open or non-host-associated) habitats. The high plasticity of the genome of this bacterial species gives it a tremendous capacity to evolve due to the gain and loss of genes through genetic changes, leading to the emergence of pathogenic strains from the commensal strains [18,19,20,21]. Genomes of pathogenic *E. coli* strains are generally larger, as the pathogenic strains require additional adaptive features, including virulence factors. Often, virulence genes are located on transmissible genetic elements such as pathogenicity islands (PAIs), bacteriophages, insertion sequences (ISs), integrons, plasmids, or transposons [22,23,24]; hence, they can also be horizontally exchanged and may facilitate novel rearrangements among different bacteria. In contrast, commensal bacteria can also become pathogenic by the loss of genes. For example, *Shigella* became virulent by the loss of *E. coli*-specific genes—such as *cadA* and flagellar genes [25,26]. Shiga toxins, enterohemolysin, cytolethal distending toxins, superoxide dismutase, and some outer membrane proteins (OMPs) are some examples of virulence factors encoded by phages in *E. coli* strains [27,28,29]. The horizontal transfer between different strains favors diversity and versatility, resulting in the creation of new pathogenic strains as well as the dissemination of acquired virulence genes with novel functions outside their clonal lineage. EHEC acquired *stx* genes (transfer by phages) [30], OI (O-island), and LEE (locus of enterocyte effacement) (PAI) through horizontal gene transfer [31]. EPEC emerged after the acquisition of the LEE island and *espC* serine protease gene [24]. Likewise, there are many identified PAIs in different *E. coli* pathotypes which were required via horizontal gene transfer and can contribute to fitness and the colonization of different niches [31,32].

The accumulating genomic sequence data has led to an increased understanding of *E. coli* virulence factors and mechanisms underlying species diversification and the tracking of foodborne disease outbreaks. In 2011, there was a foodborne outbreak of diarrhea caused by Shiga-toxin-producing *Escherichia coli* (EHEC-STEC-VTEC) O104:H4 in Germany associated with the consumption of raw fenugreek sprouts [33]. Interestingly, this strain was more virulent than most Shiga-toxin-producing *E. coli*. DNA sequencing demonstrated that the strain contained toxin-encoding phage similar to the 933W phage found in EHEC and also harbored plasmid-borne virulence factors typically found in EAEC which promote aggregative adherence to intestinal epithelial cells [34,35].

Another example is the STEC/UPEC serotype O2:H6 ST141 strain, a STEC with virulence genes *α-hlyA*, *cnf1*, and *clb* common to UPEC [36,37]. This hybrid strain can be a melting pot for pathotype conversion of InPEC and ExPEC because it acquired the *stx*-harboring prophage and ExPEC PAI. It has been also demonstrated that ST141 includes different hybrid versions, namely, UPEC/EAEC, STEC/UPEC/EAEC, and UPEC-Stx/UPEC [38].

### 1.2. Phenomenon of Antibiotic Resistance

It has been shown that *E. coli* can be highly resistance to many of the antibiotics used by humans since the 1930s [39,40]. This may be due in part to the high rate of gene acquisition and horizontal transfer capacity of *E. coli* strains. The emergence of antibiotic resistance might be multifactorial, but it is largely believed to be caused mainly by human activity and increased antibiotic usage for human health, animal health and food production. [41,42,43,44,45]. The broadly reported multidrug-resistant *E. coli* ST131 is an example of highly virulent ExPEC associated with urinary and bloodstream infections and has promoted the widespread dissemination of the *CTX-M-15* gene [46,47]. In general, the *E. coli* strains have evolved to resist major classes of antibiotics such as β-lactams, quinolones, aminoglycosides, sulfonamides and fosfomycin. AmpC-producing *E. coli* strains are dominant in gut colonization of both animals and humans and environmental contamination in developing countries [41,48,49,50]. As ESBL- and AmpC-producing *E. coli* are increasingly reported as the cause of severe infections [51,52], we are confined to the last resource antibiotic classes such as polymyxins and carbapenems. Additionally, carbapenem-hydrolyzing oxacillinase-48 (Oxa-48) carrying *E. coli* strains have also been isolated in Europe [53] where 134 cases of *E. coli* strains carrying the OXA-48 variant OXA-244 were isolated from clinical samples in Germany [54]. This same variant was further identified in 119 *E. coli* strains isolated from other European countries [55,56,57,58,59]. Likewise, New Delhi metallo-β-lactamase (NDM-1) and closely related enzymes are a group of zinc-requiring metallo-β-lactamases capable of hydrolyzing a broad range of β-lactams including all penicillins [60], cephalosporins and carbapenems. Further, resistant strains containing New Delhi metallo-beta-lactamase 1 (NDM-1) as well as over 20 NDM variants have spread and are associated with infections in many parts of the world [61,62]. With the increase in international travel and trade globalization, resistant bacteria have become a worldwide public health threat. Similarly, there is widespread use of antibiotics in food animals for various reasons, namely, growth promotion or ongoing mass prophylactic medication. In many developing countries, there is widespread use of third- and fourth-generation cephalosporins (ceftiofur and cefquinome) and fluoroquinolones (enrofloxacin) in food animals [63,64,65]. The problem is much worse in developing countries due to the increasing number of extended-spectrum β-lactamase-producing and fluoroquinolone-resistant *E. coli* due to a lack of regulation, resources, controls, and surveillance [50,66].

Thus, to counteract the above-mentioned antibacterial resistance of highly virulent strains, global efforts are needed to ensure the discovery of alternative solutions. Currently, antimicrobial-resistant pathogens are causing 700,000 deaths per year, and 10 million deaths per year are expected by 2050, a number even higher than the 8.2 million caused by cancer today [67,68]. The development of novel therapeutic approaches will continue to be essential. In addition, alternative sustainable preventive strategies such as vaccination can help in limiting the increase of antibiotic-resistant *E. coli*. In the following sections of this review, we will summarize the vaccination strategies to prevent diseases caused by different pathotypes of *E. coli.*

## 2. Diarrheagenic *E. coli* Pathotypes

Diarrhea was responsible for the deaths of at least 370,000 children in 2019; 800,000 fatalities per year according to data from 2013, and the second leading cause of death in children under five years old [69,70]. Diarrhea is a global problem, but its high morbidity and mortality occur in low-income countries. Most of these are caused by InPEC whose members possess distinct virulence traits, different O:H serotypes and characteristic clinical syndromes even though they share some steps in the mechanism of pathogenesis that include an attachment to the intestinal mucosa and harbor plasmids that encode virulence factors [71].

### 2.1. EPEC

Among the pathotypes of InPEC, EPEC is a major cause of infantile diarrhea in developing countries, varying from subclinical to fatal infections [72]. The characteristic histopathological hallmark related to this group of *E. coli* is the production of lesions known as “Attaching and effacing (A/E)” lesions that are produced when bacteria intimately attach to intestinal epithelial cells and alter the cytoskeleton through the accumulation of polymerized actin beneath the adherent bacteria [73,74]. EPEC is divided into typical EPEC (tEPEC) and atypical EPEC (aEPEC), based on the presence of EPEC adherence factor plasmid (pEAF) [75,76]. They have their own distinct adherence patterns. aEPEC strains exhibit diffuse adherence, localized adherence, and an aggregative adherence pattern, whereas tEPEC strains display a localized adherence pattern. The aEPEC can cause both acute diseases and persistent bloody diarrhea [3]. The tEPEC strains are strongly associated with abundant secretory diarrhea with mucus and significant water and electrolyte losses [71,74]. While aEPEC can be found in both humans and animals, the reservoir of tEPEC is human [76].

#### 2.1.1. Molecular Pathogenesis

**Figure 1:**
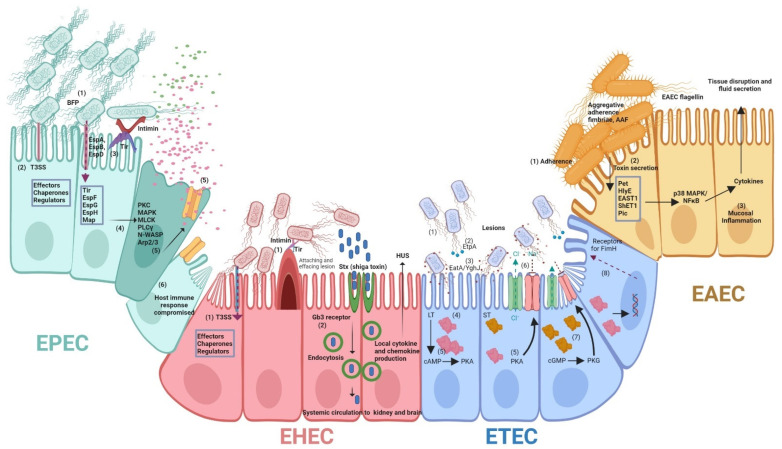
Combined pathogenesis of EPEC, EHEC, ETEC and EAEC. EPEC (Gray) is famous for production of lesions known as “Attaching and effacing (A/E)” lesions that are produced when bacteria intimately attach to intestinal epithelial cells. Bundle-forming pilus (BFP) helps in interbacterial adherence as well as adherence to epithelial cells (1). Pathogens contact the host cell via T3SS and its effectors (2). The intimate attachment of bacteria with the epithelial cells is mediated by the Tir-EspA-EspB-EspD complex into the host membrane and intimin on the bacterial membrane (3). The type III secretion system releases various effector proteins—including Tir, EspF, EspG, EspH and MAP are migrated in the cytoplasm (4) where it interacts with host proteins such as N-WASP and the Arp2/3 complex to cause actin rearrangement and the pedestal formation. Protein kinase C (PKC), phospholipase Cγ, myosin light-chain kinase and mitogen-activated protein (MAP) kinases are triggered. These multiple complex effacements lead to increase intestinal inflammation, intestinal permeability, and loss of absorptive surface area (5,6). EHEC (Red) is also attaching and effacing (A/E) pathogens that efface the microvilli and subvert host cell actin to form pedestals beneath the attachment site, but the mechanism is slightly different from EPEC, Tir is not phosphorylated. EHEC injects effector proteins such as Tir and EspFu into the host cytoplasm through the T3SS (1) and Tir binds to intimin to attach the bacteria to the host cell (1). Tir and EspFu recruit host factors to subvert host cytoskeleton and actin polymerization. In addition, Shiga toxin (Stx; also known as verocytotoxin) is released in response to stress, further contributing to disease. The B subunit of Stx toxin binds to the glycosphingolipid globotriaosylceramide (Gb3), present in lipid rafts on the surface of the target cell and is internalized (endocytosis) (2), and the Shiga toxin is activated through cleavage of the A subunit into two fragments by the protease furin. Stx toxin, if absorbed into the systemic circulation, can cause direct endothelial injury by increasing inflammation, inducing expression of cytokines and chemokines, and even can damage important organs, especially the kidney and the brain. ETEC (Blue) strains adhere to intestinal epithelial cells with the help of one or more peritrichous flagella or fibrillar colonization factors (CFs) (1). EtpA mediates the bridging of flagella with glycan receptors present in mucin (2). Mucinolytic serine protease EatA helps to degrade MUC2, the major mucin secreted by goblet cells, while YghJ is required for efficient access to the surfaces of enterocytes (3) providing bacterial access to the epithelial surface. Coincident with these events, the bacteria deliver pre-formed LT and ST to their respective receptors on the host cell GM1 gangliosides and GC-C, respectively (4), which activates the production of cellular cyclic adenosine monophosphate (cAMP) (5), that initiates intracellular signaling cascades that ultimately lead to chloride efflux from CFTR and inhibition of Na+ uptake through the NHE3 Na+/H+ ion exchanger; resulting in the net export of salt and water into the intestinal lumen and diarrhea (6). Heat-stable toxin (ST) binds to guanylate cyclase C to activate the production of cyclic guanosine monophosphate (cGMP), activating protein kinase G (PKG), which phosphorylates ion channel proteins and the development of diarrhea (7). LT modulates the transcription of multiple genes including those encoding CEACAMs (Carcinoembryonic Cell Adhesion Molecules), which then serve as receptors for FimH of ETEC expressing type 1 fimbriae that promote ETEC adhesion (8). EAEC (yellow) adheres to small and large bowel epithelia in a thick biofilm, releases enterotoxins and cytotoxins, and induces mucosal inflammation. The first step of EAEC infection involves colonization of intestinal mucosa by aggregative adherence fimbriae (AAF) I, II and III which gives characteristic AA pattern as a stacked-brick lattice (1). This is followed by the secretion of different enterotoxins and cytotoxins with different functions, namely, Enteroaggregative heat-stable toxin (EAST-1), Plasmid-encoded toxin (Pet), Protein involved in colonization (Pic), Shigella enterotoxin 1 (ShET-1), and Hemolysin E (HlyE) (2). The role of these virulence factors and their clinical outcome is unclear, but they are associated with increased cytokine production and inflammatory markers resulting in mucosal inflammation (3). The figure was created with BioRender.com (accessed on 22 January 2023).

EPEC pathogenesis involves three steps: (i) initial adherence to host cells, (ii) translocation of bacterial toxins using a type III secretion system, (iii) pedestal formation and intimate attachment [3,74,77] (Figure 1). At first, EPEC strains adhere to the enterocytes in the small intestine and form microcolonies in localized or aggregative patterns. This adherence is fostered mostly by a plasmid-encoded, bundle-forming pilus (BFP). It has been shown that BFP mutant strains are less able to cause diarrhea in human volunteers [78,79]. Initial adherence and microcolony formation are important for EPEC infections, and BFP is one of the key virulence factors in pathogenesis [80,81,82].

The locus of enterocyte effacement (LEE), also found in EHEC, encodes a type III secretion system (TTSS), translocators (EspA, EspB and EspD), effectors (Tir, EspG, EspF, Map and EspH), chaperones (CesAB, CesD, CesD2, CesF, and CesT) and regulators (Ler, GrlA, and GrlR). TTSS generates a pore to translocate these proteins from bacterial cytoplasm to the outside environment [71,74,77,83]. It has been shown that Esp translocators are necessary for A/E lesion formation. EspA mediates the translocation of EspB and EspD into host cytosol by making filamentous appendages surrounding the bacterium and forming a translocation tube that interacts with host cells. Rather than acting as translocated effectors, EspB and EspD primarily function to deliver virulence proteins to host cells [84,85].

During the last stage of EPEC infection, pedestal formation, enterocyte effacement and intimate bacterial attachment to host cells take place. The model for EPEC pathogenesis suggests that EPEC initially adheres to epithelial cells by the interaction of intimin with the translocated intimin receptor (Tir) inserted in the membrane [86]. Subsequent effacement of microvilli leads to loss of absorptive surface area, increased intestinal inflammation, and fluid accumulation in the intestinal lumen [87,88]. In addition, some of the EPEC strains produce an enterotoxin, EspC which is a serine protease autotransporter. EspC has no role in the generation of EPEC A/E lesions but controls pore formation and cytotoxicity by cleaving EspA/EspD which are the translocator components of the Type III secretion system (T3SS) [89]. It is interesting to note that EspC could contribute to EPEC infection by degrading different biological substrates such as pepsin, glycoprotein, coagulation factor V, and spectrin) and by inducing apoptosis and necrosis in epithelial cells [90].

#### 2.1.2. Vaccine Strategies against EPEC

The aforementioned virulence factors which are important for the pathogenesis of EPEC are potential immunogens to stimulate intestinal immune responses and possible targets as vaccine candidates. One report has shown that recombinant *Mycobacterium smegmatis* (Smeg) and *Mycobacterium bovis* BCG strains expressing BfpA or intimin were able to trigger the immune response in mice [91]. It is hypothesized that the binding of secretory IgA antibodies to BFP may interfere with bacterial binding or initial attachment of EPEC and may prevent the downstream signal transduction pathway to manifest diarrhea [92,93,94]. Spleen cells grown in vitro from recombinant BfpA-immunized mice produced TNF-α and INF-γ. TNF- γ is produced by recombinant intimin [83,95]. A successful immunization of cattle against an EHEC (*E. coli* O157) strain has been performed with a combination of recombinant EspA, intimin and Tir. In addition, humoral and cellular immune responses are triggered in mice immunized with *Lactobacillus casei* strains constructed to express intimin-β fragments (*L. casei*-Int_cv_) and immune dominant epitopes of Int280 [96]. These collectively signify virulence factors alone, or in combination with non-infectious immunogenic vectors, can work synergistically to protect against EPEC diarrhea [83]. A list of vaccine projects in different stages against EPEC is listed in Table 1.

### 2.2. EHEC

EHEC strains are Shiga-toxin encoding *E. coli* (STEC), also called verotoxin-producing *E. coli* (VTEC) that infect the alimentary tract and cause hemorrhagic diarrhea [102], hemorrhagic colitis and hemolytic-uremic syndrome (HUS) that can result in kidney failure and neurological complications in humans [103,104]. Unlike EPEC which predominantly infects the small intestine, EHEC colonizes the large intestine [105]. EHECs are mainly responsible for severe cases of foodborne infection out of >200 known serotypes of EHEC, whereas O157:H7 have been associated with most foodborne outbreaks [106,107]. It was first isolated from undercooked minced meat after a multi-state outbreak in 1982 in the United States [108]. Because of multiple outbreaks, easy transmission and complications for the use of antibiotics for EHEC treatment [109,110], this pathotype represents a major public health issue [111,112,113,114,115]. EHEC naturally resides in the intestine of ruminant animals, and zoonotic transmission occurs after the consumption of contaminated animal products (mainly ground beef), contaminated water (cross-contaminated from beef), improperly cooked meats, and inefficiently washed fruits and vegetables [102,103,108]. In the United States, an estimated 63,000 cases of hemorrhagic colitis caused by EHEC are reported annually [116]. HUS is a leading cause of acute renal failure in children for the past two decades [117,118]. While there is an increased burden of EHEC outbreaks mainly affecting developed countries, an EHEC-EAEC hybrid strain also caused a serious outbreak in Europe, with 3816 reported cases that included 54 deaths and 845 HUS cases [119,120].

#### 2.2.1. Molecular Pathogenesis

Stx toxins, also known as verotoxins (VT), are the key virulence factor of EHEC. The Shiga toxin family is composed of Stx1 which is nearly identical to the Shiga toxin of *Shigella dysenteriae* and differs only at a single amino acid whereas Stx2 shares less than 60% amino acid homology with Stx1 [102,103,121]. Stx consists of five identical B subunits that are soluble toxins [108]. After bacteria release these powerful cytotoxins, they are translocated from the gut lumen to nearby tissues and the bloodstream. The holotoxin binds to the glycolipid globotriaosylceramide (Gb3) on the target cell surface, and the toxin is internalized where a single A subunit cleaves ribosomal RNA and halts host cell protein synthesis. Stx can also induce apoptosis in intestinal epithelial cells and can cause local damage in the colon resulting in hemorrhagic colitis, necrosis, and perforation of the intestine [122,123]. EspP is a serine protease autotransporter, which contributes to biofilm formation by forming macroscopic rope-like polymers that are refractory to antibiotics and mediate adherence to host cells and cytopathic effects [124]. EspP can also cleave host coagulation factor V and serpins which can result in prolonged hemorrhage and contribute to EHEC pathology [125,126,127].

#### 2.2.2. Vaccine Strategies against EHEC

In the past two decades, EHEC has been the most studied *E. coli* pathotype for vaccine development mainly due to the severity of infection with an increased number of HUS cases, and issues preventing the use of certain antibiotics to treat STEC [109]. Unlike other pathotypes, the use of antibiotics exacerbates STEC disease as antibiotics can induce EHEC to increase the production of Shiga toxins and release more toxins during bacterial cell lysis. Since EHEC can colonize the intestine of animals, there have been successful experiments to assay the colonization ability of EHEC in different animal models ranging from neonatal calves [128,129], germ-free piglets (where they also show CNS symptoms such as severe infection in humans) [130,131], ferrets (oral infection model) that develop HUS following O157 infection, to different murine models (intra-gastric inoculation model) [132,133,134,135]. The availability of a wide range of animals provides valuable models to investigate host immune responses following EHEC infection as well as for vaccine development.

To date, different strategies such as Shiga toxin-based vaccination to neutralize the effect of toxin by using antibodies engineered against the A subunit of Stx2 and B subunit of Stx1, Stx toxoids [136,137], attenuated bacteria- where the LEE-encoded regulator (Ler) which regulates genes inside, and outside pathogenicity island region is deleted or disrupted making the bacteria non-pathogenic but immunogenic enough to impede the pathologic effects of the toxin [138]. In addition, other methods such as bacterial ghosts where bacteria are engineered to have controlled expression of a lysis gene that lyses the bacteria and forms empty bacterial cell envelopes with the composition of the cell envelope of living cells which are non-infectious but induce the mucosal immune response. Other protein-based, peptide-based, plant-based, DNA-based, polysaccharide-based, or adjuvant-enhanced vaccines have been reported to induce the host immune response and decrease the lethality of EHEC in different animal models, and are summarized in Table 2 [71].

The attaching and effacing (A/E) family of gastrointestinal bacterial pathogens includes EPEC and EHEC. Both EPEC and EHEC have a high homology in their LEE and O islands [139]. Virulence of these pathotypes depends on a T3SS and equivalent secreted proteins such as EspS and intimin [71]. From epidemiological data, it has been observed that EHEC occurrence is significantly lower in the region where EPEC is endemic [140,141]. This strategy has been exploited by researchers to utilize the attenuated EPEC O126:H6 where EspB and intimin antibodies from EPEC vaccination were cross-reactive with EspB and intimin from EHEC and showed reduced mortality in mice following an EHEC infection [140]. These vaccines are summarized in Table 2.

**Table 2:** Reports describing vaccines against Enterohemorrhagic *E. coli* (EHEC).

Type of Vaccine	Component of Vaccine	Results/Observations/Outcomes	Animal Model (Year)	References
Attenuated bacterial vaccines	Attenuated *Salmonella enterica* Typhimurium expressing recombinant EspA, intimin and Stx2B.	Significantly higher antibody titers against EspA, intimin and Stx2B, and specific lymphocyte proliferation.	Mice immunized orally (2011).	[142]
	γ-intimin variant expressed by attenuated *Salmonella enterica* Typhimurium χ3987 (Δ*cya,*Δ*crp,*Δ*asd*) and H683 (Δ*aro*Δ*asd*).	Increased IgG in serum and IgA in feces of mice. Reduced EHEC O157:H7 shedding and colonization post-challenge.	Oral immunization of mice (2012).	[71]
	Attenuated EPEC O126:H6.	Reduced mortality in EHEC challenged mouse model and cross-reaction against EspB and intimin EPEC antibodies with EspB and intimin from EHEC.	Mice immunized orally (2016).	[143]
	Recombinant bacillus Calmette-Guérin expressing Stx2B (rBCG-Stx2B).	Significant levels of Stx2 IgG in mice. Higher survival rate (>65%) of immunized mice challenged with EHEC.	Mice immunized orally (2012).	[144]
	EHEC O157:H7 86-24 strain Δ*ler*Δ*stx2* expressing Stx1A Stx2A detoxified.	Lower colonization of EHEC O157:H7 after challenge.	Oral immunization of mice (2009).	[138]
Shiga toxin-based vaccines	cαStx1B and cαStx2A antibodies.	Safety and good tolerance in a human trial single-dose study.	Human volunteers (2009).	[136]
	one anti-serum albumin VHH and two copies of anti-Stx2B VHH.	Decreased toxicity of EHEC in Stx2 lethal mouse model.	Mice immunized orally (2016).	[137]
Bacterial ghost-based vaccines	Bacterial ghosts of O157:H7 which is unable to cause infection.	Anti-toxicity effect on Vero cell culture. Reduced colonization of EHEC O157:H7 and 93% and 100% survival in orally and rectally immunized mice, respectively.	Orally and rectally immunized mice (2015).	[145]
	Stx chimeric protein exposing bacterial ghosts of O157:H7 (Stx2Am-Stx1B).	Increased IgG and IgA antibody titers to Stx1A and Stx2B. Survival rate >50% in immunized mice.	Intranasal immunization of mice (2012).	[146]
Peptide-based vaccines	Peptide KT-12 (KASITEIKADKT) conjugated with KLH.	Elevated levels of IgG in subcutaneously immunized mice and IgA in intranasally immunized mice.	Intranasal immunization of mice (2011).	[147]
	C-terminal region of intimin.	Reduced bacterial adherence to Hep-2 cells and confers protection in immunized mice.	Oral immunization of mice (2011).	[148]
Protein-based vaccines	EspA-Stx1A fusion protein-based vaccine.	Crude toxin Stx2-challenged mice showed 95% survival with high titers of IgG to EspA-Stx1A in treated mice.	Oral immunization of mice (2009).	[149]
	Stx1B-Stx2-truncated intimin fusion protein.	EHEC O157:H7 challenged immunized mice had a 100% survival rate.	Mice model (2009).	[71]
Plant-based vaccines	Cell line from *Nicotiana tabacum* (tobacco) NT-1 that expresses inactivated Stx1A.	Stx2-specific IgA in feces of orally immunized mice, and protection against STEC with more than 75% survival rate.	Orally immunized mice model (2018).	[71]
	Five recombinant EHEC proteins, including NleA, Stx2b, and EspA expressed from *Nicotiana benthamiana* and transplastomically in *Nicotiana tabacum*.	Immunized sheep with leaf tissue (feeder) showed less shedding of EHEC O157:H7 when challenged.	Sheep (2018).	[150]
Adjuvant improved vaccines	Adjuvanted EspB and/or C-terminal of γ-intimin protein with MALP-2.	Significantly higher titers of IgA in immunized mice.	Orally immunized mice (2013).	[151]
	Chimeric Tir-Stx1B-Stx2B adjuvanted with Zot.	Significant increased IgA and IgG and reduced bacterial shedding in feces post- challenge in subcutaneously immunized mice. Partial protection against EHEC.	Subcutaneously immunized mice (2019).	[152]
Polysaccharide-based vaccines	O-specific polysaccharide of EHEC O157:H7 conjugated with recombinant exotoxin A of *P. aeruginosa.*	Elevated IgG against LPS in vaccinated children with non-collateral reactions to the vaccine.	Human volunteers (2014).	[153]
DNA-based vaccines	Stx2AΔAB DNA vaccine.	Immunized mice showed partial protection when challenged with native Stx2. Toxin neutralization is observed in the Vero cell culture.	Intranasally immunized mice (2009).	[71]
	C-terminal domain of *EscC.*	Increased IgG in sera and IgA in feces of immunized mice. Reduced bacteria in feces, colon, and cecum post-challenge with EHEC.	Orally immunized mice (2014-2016).	[154,155]
	pVAX-efa1 (*efa-1*′).	Significantly elevated levels of specific mucosal IgA and reduced EHEC colonization post-challenge.	Intranasally immunized mice (2016).	[156]

### 2.3. ETEC

The ETEC pathotype is one of the principal causes of acute “travelers’ diarrhea”, affecting tourists visiting low-income countries, and is predominant in areas with poor sanitation and inadequate clean water [157,158]. ETEC infections are often characterized by diarrheal illness ranging from mild and self-limiting to cholera-like symptoms [159]. They are defined by the production of heat-labile (LT) and heat-stable (ST) enterotoxins, which disrupt secretion mechanisms in the intestine, leading to watery diarrhea [160,161]. Globally, there are millions of cases of ETEC infection and tens of thousands of deaths annually in developing countries among children less than 5 years old [162,163]. ETEC infections are also a major cause of traveler`s diarrhea with more than one million annual cases worldwide. In the United States alone, there are 40,000 estimated cases of ETEC infection annually [164,165,166].

#### 2.3.1. Molecular Pathogenesis

ETEC strains adhere to the small intestinal mucosa with the help of one or more proteinaceous pili/fimbriae also called colonization factors (CFs) [167]. Following initial adhesion and colonization, ETECs cause diarrhea not by invading the mucosa but by producing plasmid-encoded heat-labile (LT) and/or heat-stable (ST) enterotoxin (Figure 1). LTs are closely related in structure and function to cholera enterotoxin produced by *Vibrio cholerae* [168]. LTs increase host intracellular cAMP through the activation of a cAMP-dependent kinase and activate the main chloride channel of epithelial cells, resulting in increased chloride secretion from crypt cells. This ion imbalance causes an alteration in electrolyte homeostasis, resulting in loss of fluids from tissue and subsequent diarrhea [169]. ETEC is also an important veterinary pathogen associated with post-weaning diarrhea in both pigs and cattle, and STb toxin is involved in diarrhea [169,170]. ETECs also secrete plasmid-encoded virulence factors EatA and EtpBAC. EatA is a serine protease autotransporter of Enterobacteriaceae (SPATE) which can cleave substrates identified for cathepsin G [171] and EtpBAC is an extracellular adhesin which is a 2-partner secretion system responsible for the export of EatA. The *yghJ* gene present in the chromosome upstream of genes encoding the type II secretion system of multiple ETEC isolates encodes another antigen YghJ (SslE). EatA and YghJ both degrade MUC2 mucin secreted by goblet cells of small intestinal epithelia and facilitate bacteria to overcome the mucin barrier and release ETEC enterotoxins upon host enterocytes (Figure 1).

#### 2.3.2. Vaccine Strategies against ETEC

Because of the substantial morbidity and mortality in the pediatric population and the long-term consequences of enteric infections on child growth and development, vaccination could be a promising means of ETEC prevention. In the past two decades, different strategies were developed and employed for ETEC vaccine development either to prevent attachment of ETEC to the proximal small intestinal mucosa or to protect via immunity, through IgA antibodies directed against LT [71,162]. Among these strategies, ETVAX is the most advanced candidate which is currently in Phase 2b clinical trials [172]. This strategy utilizes inactivated whole cell (one *E. coli* K-12 and three O78 positive *E. coli*) vaccine strains that overexpress CFA/I, CS3, CS5 and CS6 antigens combined with hybrid LT/CTB (B subunit BS of cholera toxin) with and without dmLT adjuvant [173,174]. ETEC strains are heterogenous, exhibiting different O:H serotypes and to provide broad spectrum protection, various fimbrial antigens present in the most prevalent ETEC pathotypes have been used in this strategy. This vaccine demonstrated excellent safety in an age-descending trial in Bangladesh, and the inclusion of the dmLT adjuvant increased the magnitude and kinetics of mucosal antibody responses in both Bangladeshi infants and Swedish adults [172]. Theoretically, a multivalent ETEC vaccine which can express CFA/I, CFA/II and CFA/IV antigens can protect against the majority of ETEC strains worldwide [175,176,177].

Another vaccine that is further along in the development is live attenuated *E. coli* expressing ETEC fimbriae. *E. coli* strain E1392-75-2A is a prototype demonstrated in clinical trials that expresses CS1 and CS3 fimbriae but lacks genes that encode LT and ST [178,179,180]. This strain was derived in the Central Public Health Laboratory, London, U.K., wherein the genes encoding LT and ST were spontaneously deleted from the CFA/II plasmid. All volunteers who received 10^10^ CFU doses of strain E1392-75-2A developed significant rises in intestinal fluid SIgA antibody to CS1 and CS3 and the vaccinees were significantly protective (*p* < 0.005, 75% vaccine efficacy) against ETEC challenge strain E24377A (O139:H28) [181]. However, around 15% of the subjects had mild diarrhea after taking this live oral vaccine. On the same lines, live attenuated vectors such as *Shigella* and *Salmonella* have been used to express ETEC fimbrial antigens and LT antigens [126,182]. An attenuated *Shigella* strain expressing ETEC CFA/I and CS3 fimbriae elicited SIgA mucosal antibody responses to those antigens in a guinea pig model [183,184]. A multivalent live oral vaccine against both *Shigella* and ETEC is being developed and is in Phase 1 clinical trial known as a ShigETEC toxin hybrid which is an LPS-free cell expressing conserved ETEC and *Shigella* antigens [185]. Likewise, a *Shigella* hybrid (1208S-122) is also in the Phase 1 trial which is an attenuated *Shigella* vaccine strain engineered to express ETEC CF and LT [162]. If this attenuated *Shigella* expresses ETEC fimbrial colonization factors and genetically detoxified LT, protection is undeniable. Further, a fimbrial tip adhesin-based vaccine, a multiple epitope fusion antigen (MEFA) based vaccine and protein-based vaccines against ETEC virulence factors are also in various preclinical phases of testing [71]. These different ETEC vaccine candidates are listed in Table 3.

### 2.4. EIEC

EIEC strains show similar genetic, biochemical and pathogenic characteristics to *Shigella,* for example, the ability to invade the mucosa of the colon [200,201]. Unlike other pathotypes such as EPEC, EHEC, and ETEC that replicate and infect extracellularly; EIEC strains penetrate the mucosa after invading epithelial cells, and can replicate and migrate within epithelial cells and macrophages as well. A large (>200 kb) virulence plasmid (pINV) found in EIEC and all *Shigella* species encodes genes required for pathogenesis [26,200,202]. This pathogenesis is known to be the result of multiple effects of the plasmid-borne type III secretion system encoded by the *mxi-spa* locus that secretes invasin proteins including IpaA, IpaB, IpaC and IpgD for signaling events and other actions [3,203]. Although EIEC infections are reported in countries with poor sanitation, hygiene and socioeconomic status, there have been EIEC outbreaks in the USA, Japan, Israel and recently in Italy in 2012 and Nottingham, U.K. in 2014 [204,205,206,207]. Most of these outbreaks are sporadic and travel related. These outbreaks signify the ability of EIEC to induce gastrointestinal disease outbreaks across the world.

#### 2.4.1. Molecular Pathogenesis

**Figure 2:**
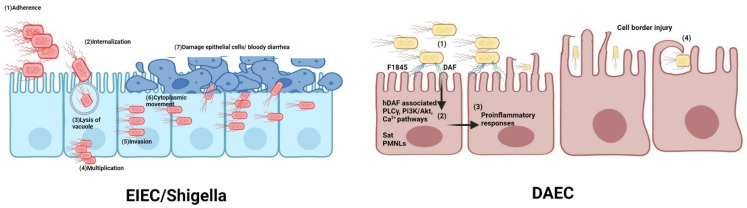
Pathogenesis of EIEC and DAEC. *Shigella*/EIEC show similar types of pathogenesis. EIECs adhere to the basolateral surface of enterocytes and form a pore in the host membrane (1). Invasion plasmid antigens (IPAs) mediate the uptake of EIEC into enterocytes (2), which is followed by a process of host cell entry (3), multiplication (4) and intercellular spread of EIEC (5,6), ultimately leading to death of the enterocyte (7). Following escape from the entry vacuole (3), Shigella/EIEC drives actin polymerization at one pole through IcsA-dependent recruitment of N-WASP and ARP2/3. This allows for intracellular motility. Invasive bacteria released from damaged enterocytes induce another wave of uptake by phagocytosis in healthy cells and then repeat the cycle of survival and replicate or disseminate from cell to cell via an actin-based motility process. DAECs are characterized by the diffuse adherence pattern on cultured epithelial cells. Around 75% of DAEC harbor adhesins from the Afa/Dr family, responsible for this adherence phenotype. The infection starts with the interaction of Afa/Dr family of adhesins with membrane-bound receptors, including decay-accelerating factor (DAF) by Afa/Dr/DAF adhesins (AfaE-I, AfaE-II, AfaE-III, AfaE-V, Dr, Dr-II, Nfa-1, and F1845) (1). A signaling pathway involving protein tyrosine kinase(s), phospholipase Cγ, phosphatidylinositol 3-kinase, protein kinase C, and an increase in [Ca2+] that controls the rearrangements of brush border-associated F-actin induce structural changes of microvilli (2). Sat, serine protease toxin secreted by DAEC can change the paracellular permeability, resulting in fluid accumulation in the intestine. Host interactions with Afa/Dr adhesins can induce migration of polymorphonuclear leukocytes (PMNLs) (3) which promotes the production of proinflammatory cytokines which in turn promote the upregulation of DAF (3). It was reported that finger-like projections are induced in enterocytes due to signal transduction caused by DAEC (4). The figure was created with BioRender.com (accessed on 22 January2023).

During infection, EIEC strains initially penetrate and enter into epithelial cells [77], lyse the endocytic vacuole, multiply inside the cells and migrate to adjacent cells through the cytoplasm (Figure 2). During initial penetration, T3SS inserts a pore containing IpaB and IpaC into host cell membranes [77]. IpaC induces actin polymerization and activates GTPases Cdc42 and Rac which leads to the formation of cell extensions. Actin polymerization and lamellipodial extensions of host cells are induced by IpaC. In the same line, IpaAs alter cell extensions induced by IpaC by binding to vinculin and inducing actin depolymerization, leading to a structure that mediates bacterial cell entry. IpgD helps the internalization by inducing host cell membrane blebbing [77,207,208,209]. Multiple factors including T3SS effectors such as IpaB and IpaC, which are used for phagosomal escape by bacteria; host factors such as Rab5 and Rab11; and other cytosolic access factors utilized by intracellular bacterial pathogens are involved in rupturing the endocytic vacuole in less than 10 min following cell entry [3,71,209]. After this, bacteria multiply inside cells and invade and damage surrounding epithelial cells that results in Shigellosis-like symptoms such as watery diarrhea with mucus, with leukocytes and blood in the stool, abdominal pain with cramps and tenesmus, fever, and systemic toxicity as well [209,210,211].

#### 2.4.2. Relationship with Shigella

In the past, enteroinvasive strains that cause shigellosis were divided into EIEC and *Shigella* species (with four subgroups: *S. dysenteriae, S. flexneri, S. boydii and S. sonnei*). Genetically, all these groups are so closely related they could be considered as the same genus or even species comprising other pathogenic and commensal *E. coli*. Like *Shigella*, EIEC strains are usually nonmotile, lysine decarboxylase negative, and commonly lactose negative [212,213,214]. The biochemical similarity between EIEC and *Shigella* is characterized by their ability to utilize serine, xylose or sodium acetate and ferment mucate [214,215]. It has been reported that non-motile serotypes of EIEC produce unusually large flagellin assembled into functional flagellum filaments [216]. EIEC/Shigella utilizes similar modes of action to manipulate host innate and adaptive immunity for penetration, multiplication and replication in host epithelial cells [3,71,209,210,214,217]. Two main features of EIEC and *Shigella* are evolution, requiring both the gain of virulence genes and the loss of function or deletion of other genes [25]. In *S. flexneri*, approximately 200 genes were acquired, and 900 genes have been lost during the divergence from commensal *E. coli* [218,219]. Some of the examples include loss of outer membrane protease (OmpT) that interferes with the localization of the actin nucleator IcsA, which is necessary for the invasion phenotype; loss of lysine decarboxylase that catalyzes cadaverine production, which inhibits enterotoxins of *Shigella* [25,220]; and the absence of functional flagella and fimbriae that are active activators of the host innate immune response, and might interfere with the initial colonization process [221,222](other genomic regions “black holes” lost by EIEC and Shigella are reviewed in detail in [214]). Due to the similarities shared between EIEC and Shigella, virulence factors responsible for EIEC or *Shigella* infections and various subtypes of attenuated Shigella strains can be exploited to devise a potential vaccine against these enteroinvasive pathotypes.

#### 2.4.3. Vaccine Strategies against EIEC/Shigella

EIEC/*Shigella* does not have any animal reservoir. They are usually transmitted among the human population through poor hygiene and sanitation. Licensed vaccines are currently not available against EIEC/*Shigella* and basic hygiene and proper sanitation remain the best way of preventing infection [223,224]. However, these approaches are not feasible in many low-income countries. Therefore, there is an increased demand for vaccine development, and strategies include the utilization of a live-attenuated strain, glycoconjugate-based candidates, novel antigen candidates such as *Shigella* outer membrane vesicles (OMV) encapsulated in nanoparticles, and protein subunit candidates [224,225,226,227,228,229]. There are several animal and human studies that have described the potential for vaccination using invasion plasmid antigens (IPAs), *virG* or *Shigella* O antibodies that cross-react with EIEC O antigens [230,231]. Table 4 summarizes vaccine approaches against EIEC/*Shigella*.

### 2.5. EAEC and DAEC

EAEC is the cause of several diarrheal outbreaks worldwide and is mostly associated with mildly inflammatory diarrhea in young (< 2 years old) and malnourished children; persistent diarrhea in HIV-infected adults and children and acute diarrhea in travelers in both developing and industrialized countries. It was first described in 1987 based on an auto-aggregative “stacked-brick” adherence pattern to Hep-2 cells in culture [238]. This phenotype is mediated by the aggregative adherence fimbriae (AAF) and aggregative adherence regulator [239,240,241].

#### 2.5.1. Molecular Pathogenesis

EAEC infection involves the colonization of bacterial cells in dense clusters to the intestinal mucosa [238,240] followed by the secretion of EAEC heat-stable enterotoxin (EAST1) and ShET1 (*Shigella* enterotoxin 1) which causes a loss of fluid [242,243] (Figure 1). EAEC also produces a plasmid-encoded SPATE autotransporter enterotoxin called Pet [244]. Pet has enterotoxic activity and induces cytoskeletal changes and epithelial cell rounding due to the breakdown of fodrin/spectrin in host cells [245]. In addition, EAEC produces another SPATE with mucinase activity called Pic [246], which contributes to intestinal colonization. Pic was also shown to reduce complement activation by cleaving complement cascade factors C3, C4 and C2 [247], which also induces polymorphonuclear leucocyte/neutrophil (PMN) activation and programmed T-cell death [248]. In this context, Pic activity can contribute to immune evasion and promote EAEC virulence.

Like EAEC, the DAEC pathotype is defined by its diffused adherence pattern on epithelial cells such as HeLa or Hep-2 cells in culture. More than 70% of DAEC strains produce fimbrial adhesins from the Afa (AfaE-I, AfaE-II, AfaE-III and AfaE) or Dr (F185) family that mediate the diffused adherence phenotype and pathogenesis. These fimbriae recognize DAF (Delay accelerating factor), a cell-surface glycosyl phosphatidylinositol-anchored protein, as the receptor and cause dismantling of the actin network in intestinal cells, resulting in long cellular extensions and elongation and malfunction of microvilli which wrap around the bacteria [238,249,250,251,252] (Figure 2). Reports have suggested the presence of virulence factors other than Afa/Dr family adhesins and they can be pro-inflammatory, suggesting a potential significance in the initiation of inflammatory bowel disease [253,254]. In addition to causing diarrhea in children, adults and the elderly [255], DAECs are also known to be associated with urinary tract infections (UTIs), pregnancy complication and asymptomatic intestinal infections in both children and adults [249,256].

#### 2.5.2. Vaccine Strategies against EAEC and DAEC

For DAEC and EAEC pathotypes, there is currently limited vaccine development. Nevertheless, bacterial adhesins are considered the best candidates, and AAF/I and AAF/II could be the possible target for EAEC. When Balb/c mice were immunized with three different modes of vaccination, namely, DNA/DNA, DNA/Protein, or Protein/Protein of AAF/I or AAF/II of EAEC, respectively, (only the DNA/Protein immunization and Protein/Protein doses of AAF/I significantly induced total IgG) [257].

Likewise, afimbrial adhesins (AFA), Dr hemagglutinin, and F1845 fimbriae are the important adhesins of DAEC and could be target antigens for vaccine development [258]. Vaccination of C3H/HeJ mice with *Escherichia coli* Dr fimbrial antigen reduced urinary tract infection due to a homologous strain bearing Dr adhesin. Immune sera with high titers of anti-Dr antibody inhibited bacterial binding to bladders and kidneys [259]. Although Dr adhesin was shown to be immunogenic in this urinary tract model, its role as an antigen in vaccination against the intestinal disease is yet to be explored.

### 2.6. AIEC

In contrast to enteroinvasive *E. coli*, Adherent-invasive *E. coli* (AIEC) is the pathotype with both abilities to adhere to and invade intestinal epithelial cells. AIEC can also survive and replicate within macrophages [3,260]. AIEC is associated with inflammatory bowel disease (IBD), Crohn`s disease (CD) and ulcerative colitis (UC) [261,262,263,264]. AIEC infections are usually characterized by diarrhea, abdominal pain, rectal bleeding, fatigue, and life-threatening complications in severe cases of AIEC and IBD. Reports of bacteriological analysis from Europe and North America showed 30–50% of Invasive *E. coli* strains are present in the ileal mucosa of Crohn’s disease patients [265,266,267,268]. However, it is still not clear whether AIEC induces intestinal inflammation resulting in IBD or whether they act as an intensifying factor by colonizing the mucosa of patients with pre-existing IBD. AIEC possesses a wide range of virulence factors.

#### 2.6.1. Molecular Pathogenesis

AIEC invades host cells in a similar fashion to other pathogens such as EIEC and *Shigella*. These strains penetrate epithelial cells by either directly invading the epithelial layer or by entering via microfold (M) cells in the epithelium of the small intestine. Following the invasion, AIEC reduces epithelial barrier function and integrity [260]. Since CD and AIEC are interrelated, most of the studies of AIEC pathogenesis were carried out in epithelial cells from CD patients. During initial adherence, it has been shown that type 1 fimbriae can bind to GP2 protein located on the apical plasma membrane of M cells [260,269]. Reports also suggest that AIEC strains expressing long polar fimbriae (LPF) adhere to M cells and mediate transcytosis of bacteria. Host glycoprotein CEACAM6 (Carcinoembryonic Cell Adhesion Molecule 6) expressed in ileal mucosa has been shown to recognize the FimH type 1 fimbrial adhesin and facilitate internalization of AIEC [270]. Outer membrane vesicles (OMVs) have a role in AIEC invasion. It has been demonstrated that outer membrane proteins (OmpA and OmpC) promote the fusion of OMVs with the Gp96 receptor expressed on the surface of epithelial cells and mediate invasion [271,272]. In addition, VAT-AIEC a protease secreted by AIEC has been reported to mediate the colonization of bacteria in a murine intestinal infection model [273]. Following the invasion, AIEC strains can replicate in host cells, including macrophages [274].

#### 2.6.2. Relation of AIEC and Crohn’s Disease

Crohn’s disease (CD) is a chronic inflammatory condition of the GI tract and AIEC strains have been isolated from the guts of CD and UC patients [261,264]. AIEC strains are pathobionts, which means they can reside in the guts of healthy individuals without causing any diseases. However, the host intestinal environment under certain conditions that affect the innate immune response could promote AIEC-induced inflammatory bowel diseases [275,276]. Following AIEC infection, autophagy, which is a cytosolic process, is induced in host cells to curb the activity of bacteria. During autophagy, bacteria are recognized by NOC proteins such as CD-associated nucleotide-binding oligomerization domain-containing-2 (NOD2) and form autophagosomes which further fuse with lysosomes. After fusion, the bacteria are degraded in auto-phagolysosomes [277]. In addition to NOC proteins, other proteins such as ATG16L1, IGRM, and LC3 are also known to mediate autophagy [260,261]. It has been reported that these mediators of autophagy are defective in CD and UC patients and may favor the intra-macrophagic replication of AIEC. Genetics models have reported that the knock-down of NOC proteins shows a loss of function against AIEC infections. Knock-down of *ATG16L1* and *IRGM* genes results in defective clearances of CD-associated AIEC [268,278].

#### 2.6.3. Insights for Vaccine Development

In recent years, many studies have elucidated AIEC virulence and its role in CD. Various therapeutic strategies other than vaccination to prevent AIEC infection and concurrent CD and UC have been tested. Among these, the most popular strategies are (i) the use of prebiotics/probiotics and postbiotics and chemical compounds to inhibition or exclude AIEC; (ii) the elimination of AIEC using antibiotics, bacteriocins/colicins, phototherapy and bacterial predation; (iii) activation of autophagy; (iv) nutritional interventions; and (v) fecal transplantation and combinational therapy [279,280,281,282,283,284]. One vaccine-based approach involves the use of flagellar antigens. A Summary of approaches for AIEC and IBD prevention is listed in Table 5.

## 3. Extraintestinal Pathogenic *E. coli*

Extraintestinal Pathogenic *E. coli* (ExPEC) are responsible for most urinary tract infections (UTIs) and are a common cause of bloodstream infections and neonatal meningitis in humans and respiratory tract and other systemic infections in poultry. This group of pathogens has a broad range of virulence factors and exhibits genomic plasticity. Depending on the type of infectious disease, ExPEC has been classed into uropathogenic *E. coli* (UPEC), neonatal meningitis-associated *E. coli* (NMEC), and sepsis-causing *E. coli* (SEPEC) in humans and avian pathogenic *E. coli* (APEC) in poultry. Some ExPECs that cause infections in humans share a close phylogenetic relationship and virulence genes with certain APEC strains. Studies have shown that poultry and poultry products can be a reservoir of potential ExPEC strains and could pose a health risk to humans. Both animal and human ExPEC strains were shown to be able to cause UTI or meningitis in rodent models, and human ExPECs were shown to be virulent in poultry infections as well [10,295,296,297]. As such, some ExPEC strains have clearly been shown to infect multiple host species [298].

### 3.1. Uropathogenic E. coli (UPEC)

Urinary tract infection is a very common bacterial infection, particularly in women, and UPEC strains account for up to 90% of community-acquired urinary tract infections (UTIs), 50% of hospital-acquired UTIs, and around 80% of uncomplicated [299,300]. UPEC isolates exhibit a high degree of genetic diversity due to the presence of mobile DNA segments which are scattered around the chromosome known as pathogenicity islands. The key virulence factors harbored by UPEC strains are type 1 fimbriae, P fimbriae, mannose-resistant adhesins, hemolysin, serum resistance, siderophores and K1 capsule [3,301]. Normally, UTI infection is initiated when uropathogenic *E. coli* originating from the bowel, is transferred to the urogenital region and ascends towards the periurethral area. Colonization is facilitated by adhesins such as Pap (P), type 1 and other fimbriae such as S, M, and F1C that mediate adherence to uroepithelial cells, which is an important initial step in the development of UTI. It has been shown that by virtue of type 1 fimbriae, *E. coli* can attach to mannose moieties that coat transitional epithelial cells [302,303]. Further, several toxins including hemolysin, a serine-protease autotransporters called Vat, Sat, and Pic and the cytotoxic necrotizing factor (CNF-1) can contribute to urinary tract colonization. However, these virulence factors are not always present among different subgroups of UPEC, suggesting that there can be multiple mechanisms of UPEC pathogenesis [304].

#### 3.1.1. Urinary Tract Infection (UTI)

**Figure 3:**
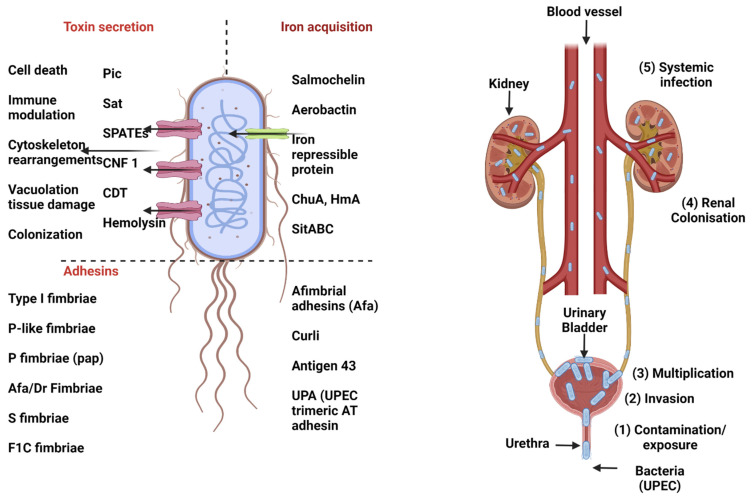
Virulence factors and pathogenesis of UPEC. UPEC possess an array of virulence factors that are distinct from those found in the intestinal pathotypes and hence allow them to colonize extraintestinal sites. Adherence is the first stage of colonization mediated by adhesins such as Type 1 fimbriae, P fimbriae, curli, and outer membrane adhesins. Each adhesin has a specific host receptor. For example, type 1 fimbriae recognize manno-oligosaccharides present on glycoprotein molecules, and P fimbriae recognize the digalactoside component of P blood group antigen present in uroepithelial cells. UPEC produce other adhesins such as Dr adhesins, F1C fimbriae and S fimbriae. Type 3 and F9 fimbriae have been associated with the formation of biofilm and catheter-associated UTIs. Iron is scarce inside the host, but UPEC can glean host iron through high-affinity siderophores including salmochelin, C-glycosylated derivatives of enterobactin, and the hydroxamate siderophore, aerobactin. The presence of heme uptake systems in UPEC helps them to acquire a readily available iron source in vivo, heme. Iron binding receptors Hma and ChuA bind to heme and the coordinated molecule is transported into the periplasm; ChuT mediates further transfer to the cytoplasm through an ATP-binding cassette (ABC) transporter. UPEC can also produce hemolysin (HlyA), cytotoxic necrotizing factor 1 (CNF 1) and autotransporter proteases such as Sat, Tsh, Vat, TagBC, and Sha. In ascending urinary tract infection (UTI), bacteria colonize the urethra and ascend to the bladder leading to cystitis (1) and sometimes subsequently the kidneys resulting in pyelonephritis (4). Bacteria adhere to the bladder surface via type 1 fimbriae which can also mediate cell invasion (2) and replicate (3) within the cell cytoplasm. When the bacteria reach the kidneys (4) there is increased risk of septicemia (5). The figure was created with BioRender.com. (accessed on 22 January2023).

The urinary tract can be affected by a variety of diseases including microbial colonization of the urine and infection of the urinary tract tissues (kidney, renal pelvis, ureters, bladder, and urethra). Clinically, urinary tract infections (UTIs) have been categorized as uncomplicated or complicated. Uncomplicated UTIs occur in the normal urinary tract of immunocompetent individuals who are otherwise healthy [305]. UTI is characterized by the presence of bacteria and neutrophils in the urine which is known as bacteriuria (presence of uropathogens in urine with more than 10^5^ CFU/mL) [306]. Bacteriuria can be asymptomatic in the host and have adverse outcomes for pregnant women and people undergoing traumatic genitourinary procedures [307]. UTI can manifest itself into cystitis (inflammation of the bladder) and acute pyelonephritis (kidney infection) (Figure 3). Cystitis is typically characterized by symptoms including frequency, burning sensation, urgency, pyuria (leukocytes in urine), dysuria, suprapubic pain and/or lower abdominal discomfort with cloudy urine. If left untreated, these infections can result in pyelonephritis which is distinguished clinically from cystitis by the presence of flank pain, fever and nausea [308]. Antibiotics are given for the treatment of symptomatic UTI but up to 25% will suffer a recurrence of infection within 6 months following treatment of the initial UTI [309]. Mounting evidence is showing that two-thirds of these recurrences are attributable to the identical initial strain recovered from a given patient suffering from uncomplicated UTIs [310,311,312].

#### 3.1.2. Molecular Pathogenesis of UPEC

The pathogenesis of UTI is complex and influenced by multiple host and microbial factors. Years of research and the use of different animal and cellular models have elucidated some of the pathogenic mechanisms. The mechanisms of UPEC infection include adherence to host cells, motility, acquisition of essential metals and other micronutrients, toxin production and evasion of the host immune response (Figure 3).

Bacterial adherence to host cells plays a key initial step in colonization and subsequent disease progression. Uropathogens must adhere to or penetrate the mucosal barrier to persist. A UTI typically initiates by contamination of the periurethra by a uropathogen from intestinal sources, followed by bacterial colonization of the urethra and bladder through filamentous adhesins known as fimbriae (pili) [313]. For example, UPEC strain CFT073, a well-characterized reference strain, encodes 12 distinct fimbrial gene clusters which code for type 1, P, F1C, Dr, Auf fimbriae as well as their chaperone and usher proteins [314]. Type 1 fimbriae have been found essential for the colonization, invasion, and persistence of UPEC in the mouse bladder. The FimH adhesin of type 1 fimbriae binds to mannosylated receptors and bladder cell surfaces known as molecules such as uroplakins, α 3 β 1 integrins and the pattern recognition receptor TLR4 [315,316,317]. The expression of type 1 fimbriae is a phase variable and is controlled by the orientation of an invertible element in the promoter region [318,319].

*E. coli* expresses another fimbria known as P fimbriae which have been associated with acute pyelonephritis in humans and showed a subtle role in pathogenesis in the murine model [320,321]. P fimbrial adherence conferred by the PapG adhesin protein binds specifically to glycosphingolipids containing digalactoside moieties found in renal epithelium and the P blood group antigen which is on the surface of some host erythrocytes [322]. So, human individuals lacking the receptor for P-fimbriae may be less susceptible to P-fimbriae-mediated adherence during UTIs caused by UPEC.

Type 1 fimbriae bind to epithelial cells and trigger a signal transduction cascade that activates the Rho family of GTP-binding proteins, resulting in cytoskeleton rearrangements in host cells and internalization of UPEC by a zippering mechanism in which the plasma membrane engulfs the bacterium [323]. This invasion benefits bacteria since the intracellular location shelters UPEC from host defenses, may reduce access to antibiotics, and prevent clearance from micturition. Although intracellular UPEC can evade host defenses, the expulsion of UPEC in urothelial cells does occur by innate immune defense through lipopolysaccharide (LPS) mediated activation of TLR4 [324]. However, a minority of internalized UPEC can escape into the epithelial cell cytoplasm and subvert expulsion and rapidly replicate exponentially in coccoid form, forming an amorphous biofilm-like intracellular bacterial community (IBC) which can cause superficial cells to protrude [325]. Later, the maturation of IBCs can lead to bacterial dispersion and the cycle of invasion of other urothelial cells [325]. Alternatively, UPEC can establish quiescent intracellular reservoirs (QIRs) in the underlying bladder cells which can later serve as seeds for UPEC release back into the bladder lumen [326,327]. Due to this phenomenon, the source of recurrent infections can be due to several sources including recurrent intestinal source contamination, vaginal colonization, or reinfection by latent bacteria within the urinary tract.

Other virulence determinants of some UPEC include hemolysin, cytotoxic necrotizing factor 1 (CNF1) and autotransporter proteins. Hemolysin (HlyA), encoded by the *hlyCABD* operon, oligomerizes and inserts into the host cell membrane in a Ca^+2^-dependent manner [328]. Hemolysin is implicated in pore formation in bladder cells and promotes their lysis and facilitates iron and nutrient acquisition [303]. Further, it can trigger cell exfoliation, apoptosis, and cytokine production and induce an inflammatory response [303,329]. CNF1 secreted by some UPEC strains alters actin remodeling and membrane ruffling, which leads to the internalization of UPEC in the host cell through the activation of Rho GTP-binding proteins: Rac1, RhoA and CDC42 [330] and may also play a role in bladder cell exfoliation [331]. In addition, activation of Rac1 and GTP induces the anti-apoptotic pathway, preventing apoptosis of the colonized uroepithelium and prolonging UPEC survival [332]. Another family of toxins that are members of the serine protease autotransporters of *Enterobacteriaceae* (SPATEs) including Sat, Pic and Vat has been characterized in UPEC. Sat (secreted autotransporter protein) can cause cytotoxic effects on bladder and kidney cells in vitro [333]; elongation of kidney cells with apparent impairment of cellular junctions [334]; degradation of fodrin and human coagulation factor V [125]; and induction of autophagic cell detachment [335]. Pic SPATE acts as a mucinase [336] and Vat SPATE can also contribute to UTIs [337,338].

To survive and compete in different host niches, UPEC also evolved multiple means of obtaining essential metals, such as iron. The most diverse and broadly distributed iron uptake mechanisms used by microorganisms are siderophore acquisition systems. Siderophores are small chelating compounds with a very high affinity for iron [339]. The direct capture of host iron is either from free heme or heme-containing proteins, such as hemoglobin. The presence of heme uptake systems in UPEC also provides a direct iron source in vivo, heme. Iron binding receptors Hma and ChuA bind to heme and transport it to the periplasm; ChuT mediates further transfer to the cytoplasm through an ATP-binding cassette (ABC) transporter [340,341]. In addition, UPEC can sequester host iron through high-affinity siderophores including salmochelins, C-glycosylated derivatives of enterobactin, and other siderophores, such as aerobactin, and yersiniabactin [342]. Salmochelins are encoded by the *iroBCDEN* gene cluster. This siderophore contributes to the virulence of ExPEC strains by escaping the action of host lipocalin-2 (siderocalin) [343]. The aerobactin siderophore is highly expressed and stable and displays a higher affinity than enterobactin at low pH [344]. Interestingly, yersiniabactin which is a confirmed siderophore also contributes to UPEC resistance to copper toxicity in urine [345]. Like iron, zinc is also an essential element for bacterial growth. The high-affinity zinc transport system ZnuACB has been expressed during UTI to acquire zinc in the host [346].

Polysaccharidic capsules and serum resistance are also important traits associated with UPEC strains, especially those which can infect the kidney and develop into systemic infections [347]. Antiphagocytic capsules can reduce the uptake of bacteria by host phagocytes and promote dissemination in blood and extra-intestinal tissues.

#### 3.1.3. Vaccine Strategies against UPEC

Generally, antibiotics are used to treat UTIs. However, increased antibiotic resistance has become an obstacle to UTI treatment [348]. Recently World Health Organization (WHO) revealed that UPEC strains causing UTI are 54.4% resistant to first-line antibiotics co-trimoxazole and 43.1% resistant to broad-spectrum ciprofloxacin [349,350,351]. Fluoroquinolone-resistant *E. coli* are widespread in the world and there are countries where Fluoroquinolone treatment is completely ineffective in more than 50% of patients [352]. To address such issues, vaccination against UTI strains might provide an important alternative to antibiotics for UTI prevention, particularly in individuals more susceptible to recurring infections.

An effective vaccine against UPEC might help reduce the current spread of infection and morbidity rate of patients as well as address the challenge of treatment of some UTIs. Effective UPEC vaccine strategies should consider the following aspects: (i) the heterogeneity of UPEC strains, (ii) the potential side effects on the commensal microbiota of the intestine and (iii) the production of multiple virulence factors by UPEC strains. Since UPEC infections involve multiple steps (Figure 3), an effective vaccine should be able to provide a protective immune response against key virulence factors required at specific stages of UTI pathogenesis, such as colonization, invasion, and the formation of IBC reservoirs. Currently, vaccines against UPEC are being developed using either cell-based or live-attenuated vaccines, killed bacteria, or antigen-based subunit vaccines against antigens such as toxins and polysaccharide-based conjugate vaccines [353].

Solco-Urovac is a whole bacterial cell-lysate-based vaccine that is multivalent and comprises cells for 10 distinct bacterial strains (6 UPEC strains, 1 strain of each *Proteus mirabilis*, *Klebsiella pneumoniae*, *Morganella morganii* and *Enterococcus faecalis*). This vaccine is known to protect against recurrent UTIs, and multiple doses reduce the prevalence of cases caused by UPEC [354,355,356]. However, this vaccine caused major side effects such as fever, burning, bleeding, vaginal itching and nausea [357]. Such deleterious side effects dismiss the use of Urovac as a vaccine against UTIs. Uro-vaxom is an oral vaccine against UTIs, which was commercialized in Switzerland in 1994, and later in other countries [358,359,360]. This vaccine comprises membrane proteins of 18 UPEC strains and is known to prevent recurrent UTIs in women. Uro-vaxom has significantly fewer side effects than Urovac; however, repeated doses taken every three months are required, which has raised issues concerning the practicality of using this vaccine in some populations of patients [361]. Other vaccines against UTIs include Urvakol and Urostim that each contain a mixture of killed bacterial pathogens including *E. coli*, *P. mirabilis*, *E. faecalis*, and *K. pneumoniae* (Urostim) and an additional *Pseudomonas aeruginosa* strain (Uravakol). These two vaccines promote immunogenicity in animals and humans, but clinical trials for Uravakol and Urostim have not been completed [362,363]. ExPEC9V is a newer vaccine which is currently in phase 3 trials (NCT04899336) and targets Invasive Extraintestinal Pathogenic *Escherichia coli* Disease (IED) in individuals aged 60 years and older with a history of UTIs [364]. In addition to this, several attempts were also made to design live-attenuated vaccines by mutating capsular or somatic O antigens of UPEC strains. For example, attenuated strains such as CP923 and NU14 Δ*waal* have been shown to produce significant humoral immune responses in mice [365,366]. However, protection from UTI has not been demonstrated yet and optimization and continued testing of such vaccine candidates are ongoing [367,368].

In other strategies, antigen-based vaccines such as capsular- or LPS-based vaccines, fimbrial and non-fimbrial adhesin vaccines, iron scavenger receptor-based, and toxin-based vaccines have been investigated as protective targets [348,369]. Currently, in a phase 2 clinical trial, ExPEC4V (NCT03500679), is a polysaccharide-based vaccine which contains O-antigens specific to serogroup O1A, O2, O6A and O25B. Two doses of this vaccine can prevent UTI even with higher bacterial doses and decrease bacteremia in women aged 18 years or older [370,371]. UPEC colonization to the bladder epithelium is promoted by adhesins which also trigger host immune responses. This principle can be utilized to develop anti-adhesin-based vaccines against UPEC. The FimH adhesin from type 1 fimbriae plays an important role in UPEC pathogenesis. It has been shown that a vaccine composed of truncated FimH or a complex of FimH with chaperone FimC (FimCH) was able to prevent the colonization of different UPEC strains in a murine model. In addition, the FimCH vaccine with Freund’s adjuvant and the FimH vaccine with Alum and MF59 adjuvants promoted an immune response against UPEC and prevented colonization in murine and primate models, respectively [372,373]. The FimH vaccine was tested in a phase II clinical trial; however, it was later rejected due to ineffectiveness in humans. Because of the variation of expression of type 1 fimbriae, UPEC strains are not consistently recognized by the immune system [353]. In addition, antigens developed against the fimbriae do not target its mannose-binding region, and differences in the expression of virulence genes in animal and human models are some of the reasons for the failure of the FimH-based vaccine. An effective UTI vaccine will potentially require multiple tests using a variety of target antigens in urinary tract models in animals [348,353]. Other vaccine candidates using FimH adhesin are currently being tested, including a TLR ligand-based vaccine where the fusion of FimH adhesin to flagellin of UPEC as a TLR5 ligand was able to induce an immune response and protected mice against UTI [374,375,376]. Moreover, a PapG fimbrial adhesin-based vaccine and Dr fimbriae with Freund’s adjuvant were also shown to generate immune responses and reduce UPEC colonization in a mouse model [377].

*E. coli* iron acquisition systems also have the same potential as UPEC vaccine candidates. It has been reported that the salmochelin receptor IroN or IroN with Freund’s adjuvant can provide protection against UTI in a murine model [378]. The aerobactin receptor IutA conjugated with cholera toxin and the yersiniabactin receptor FyuA with Alum as an adjuvant also generated strong immune responses and protection against UPEC infection in murine models [379,380]. In another study, the efficacy of a multi-epitope vaccine composed of siderophore receptors had been evaluated using an attenuated *Salmonella* vaccine delivery system, where IroN, IutA, IreA and FyuA improved protection in the urinary tract of mice [381,382]. In the same line, a toxin-based vaccine containing antigens such as hemolysin HlyA, recombinant hemolysin, or mutated CNF1 and HlyA toxins, has reduced UTI symptoms in murine experimental infection models [348,353]. Auto-transporter toxins such as Vat from UPEC strains have also been investigated as vaccine targets. In the sepsis model study, Vat improved protection by 32% and 78% after active and passive immunization, respectively, compared to unvaccinated controls [383].

### 3.2. MNEC, SEPEC

This ExPEC pathotype is associated with cases of meningitis particularly in neonates [384]. Approximately 80% of isolates from neonatal meningitis harbor antiphagocytic K1 capsular polysaccharide [385,386] and most belong to serogroups such as O18, O7, O16, O1, and O45 [387]. Inflammation of the meninges by MNEC comprises various stages of infection. Firstly, the translocation of bacteria into the circulatory system and upon reaching at least 10^3^ CFU/mL in the blood, MNEC can breach the blood-brain barrier (BBB). The meninges which form a structural and functional blood-brain barrier are composed of endothelial cells called brain microvascular endothelial cells (BMECs). Most MNEC produce S fimbrial adhesins involved in BMEC binding to the NeuAc *α*-2,3-galactose receptor [388], type 1 fimbriae and OmpA [389], which contribute to adherence and invasion of brain endothelial cells. Furthermore, an outer membrane protein, NlpI, can also mediate the binding/invasion of MNEC to brain endothelial cells [390]. The CNF1 toxin through activation of the RhoA pathway may also contribute to MNEC invasion of BMECs and penetration into the brain [391]. IbeA has also been associated with the invasion of BMEC [392]. In addition to the role of IbeA in the invasion and crossing of BBB, it has been linked to the regulation of expression of type 1 fimbriae [393] which can mediate endothelial cell adherence and invasion. Further, IbeA may contribute to oxidative stress resistance [394] and the protection of bacteria against H_2_O_2_ stress. The K1 polysaccharide capsule can contribute to serum resistance, antiphagocytic properties and intracellular survival [347] and, as such, it is an important virulence factor of most MNEC strains.

#### Vaccine Strategies against MNEC

Despite many efforts, no vaccine has yet been developed to help prevent neonatal meningitis caused by *E. coli*. The vaccine development is impeded by technical difficulties. There are well-established animal models of MNEC-induced meningitis in neonatal mice and rats to investigate MNEC pathogenesis [395,396,397], but vaccine assessment is not favorable because it takes 2–3 weeks for active immunization [398]. Since this is a devastating disease among newborns and premature infants, alternative prophylactic therapies such as passive protection antibodies can be beneficial for the control of MNEC infection [396,399]. There has been research on capsular polysaccharides (CPs) as a good candidate for vaccine development, but O-acetylated colominic acid produced by *E. coli* K1 was poorly immunogenic due to its similarity to polysaccharides found on the surface of human tissues [400]. Preventative approaches against MNEC may benefit by using virulence proteins as vaccine targets or for the generation of protective antibodies for passive immunotherapy of neonates against MNEC [401].

## 4. Pathogenic *E. coli* of Importance to Animal Health

The first section of this review is focused on *E. coli* pathotypes associated with human infections and vaccination strategies to potentially prevent pathogenic *E. coli* from causing disease in humans. Importantly, *E. coli* is also an important pathogen of livestock and poultry and can cause both enteric and systemic infections. As such, it is important to consider the pathotypes of *E. coli* associated with infections of importance to animal health and potential approaches to prevent such infections through vaccination strategies.

### 4.1. Avian Pathogenic Escherichia coli (APEC)

Avian colibacillosis is a common disease in the poultry industry worldwide. As such, APEC is a major cause of morbidity and mortality in chickens due to localized or systemic infections, resulting in heavy economic losses to the poultry industry [402]. APEC also infects other avian species including turkeys and ducks [403,404,405]. A variety of disease types have been observed: including yolk sac infection, omphalitis, swollen head syndrome, respiratory tract infection, septicemia, enteritis, and cellulitis. Colibacillosis in chickens results in death by septicemia in acute infection, whereas subacute infections can result in pericarditis, airsacculitis and perihepatitis [406]. APEC strains commonly belong to three serogroups, O1, O2, and O78 although many different serogroups have been identified in APEC and O1, O2, and O78 are not always predominant in certain studies [407]. Several virulence factors have been associated with APEC strains [408]. These include fimbrial adhesins (type 1 fimbriae [409], P fimbriae [410], and Stg fimbriae [411], curli [409], Yqi [412]; outer membrane proteins and other surface molecules contributing to serum resistance or antiphagocytic properties (Increased serum survival (Iss) [413,414]), two-component signal transduction systems (RstA/RstB [415], PhoB/PhoR [416], BarA/UvrY [417]), O78 lipopolysaccharide, and K1 capsule [418,419]; iron and metal acquisition systems (aerobactin [420], salmochelin [421], yersiniabactin [422]), heme utilization/transport protein ChuA [423] and the Sit metal transport system [424]; autotransporters (Tsh, Vat, Sha, TagB, TagC, AatA [425,426,427,428], phosphate transport and the Pho regulon (the Pst system) [429]; sugar metabolism [430]; and nitrite transporter NirC [431]. Specific virulence genes including *iss*, *iroN*, *ompT, iutA* and *hlyF* are commonly present in APEC and are frequently encoded on large plasmids such as Colicin V (ColV) plasmids [432]. In addition, other factors such as IbeA, and the type VI secretion system, which are known to affect the expression of type I fimbriae, are involved in the colonization of the respiratory tract [433]. However, no single common virulence factor has been identified in all strains.

#### 4.1.1. APEC Pathogenesis

**Figure 4:**
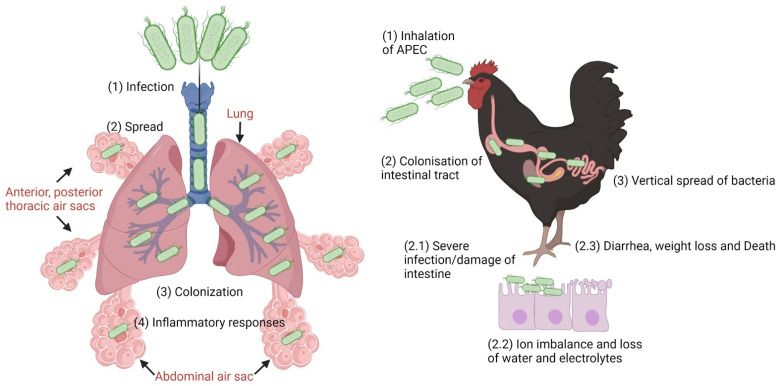
Overview of APEC infection. APEC can reach the trachea by inhalation of contaminated aerosol particles (1). Once inside lung and airsac, the avian immune response is triggered by interactions with pathogen-associated molecular patterns (PAMPS) recognized by receptors such as the Toll-like receptors (TLRs) (2). Inflammatory responses to bacterial infection will elicit macrophages and heterophils to the infected site. Inflammation will lead to tissue damage. APEC can also reach the gastrointestinal tract (2) and sometimes cause diarrhea (2.1,2.2) or invade through intestinal epithelium in presence of environmental stressors (production-related stress, immunosuppression, and concurrent infections) leading to systemic infection (2.3). APEC can be transmitted to other chickens via fecal-oral or aerosol route or vertically spread by infection of eggs (3). The figure was created with BioRender.com (accessed on 22 January2023).

*E. coli* is an intestinal commensal of poultry [434], but some of these fecal isolates when inhaled by poultry can colonize the respiratory tract and then cause colibacillosis which can include respiratory infection as well as a systemic fatal disease [435]. Colonization in the trachea and the air sacs is considered the first step of systemic infection for APEC (Figure 4). Airsacculitis is a common type of infection in poultry of all ages. In addition to the respiratory route, various other infection routes have been described: neonatal infections, infections through dermal lesions and infections of the reproductive organs. Once in the blood, APEC can then disseminate to the liver and pericardium, and the infection may also lead to bacteremia and septicemia [435]. A laying hen having *E. coli*-induced salpingitis can have an infected egg before shell formation [436]. This can lead to infection and a high mortality rate among young chicks during the first few days or weeks after hatching through yolk sac infection [436].

Adhesins such as fimbriae, Stg, Tsh and Yqi can contribute to APEC colonization of the respiratory tract: the trachea and the lungs [427]. Carbohydrate metabolism may also contribute to the colonization of the lung or air sacs [430]. To disseminate in the bloodstream, bacteria need to translocate through the lungs and air sac interstitium. K1 capsule was shown to facilitate the translocation of APEC into the vascular system [437]. When APEC disseminate in the blood, they are confronted by innate immune defenses and nutrient limitation. In this context, siderophores such as aerobactin and salmochelins, the ChuA heme uptake and Sit metal transporter sequester iron and other metals from the host environment [421]. In addition, protectins which help to evade the host immune system such as K1 capsules, Iss, Type III secretion system, and specific O antigens can contribute to survival and virulence through resistance to the avian immune cells and the serum complement system [418].

#### 4.1.2. Vaccines and Vaccination Strategies against APEC

**Figure 5:**
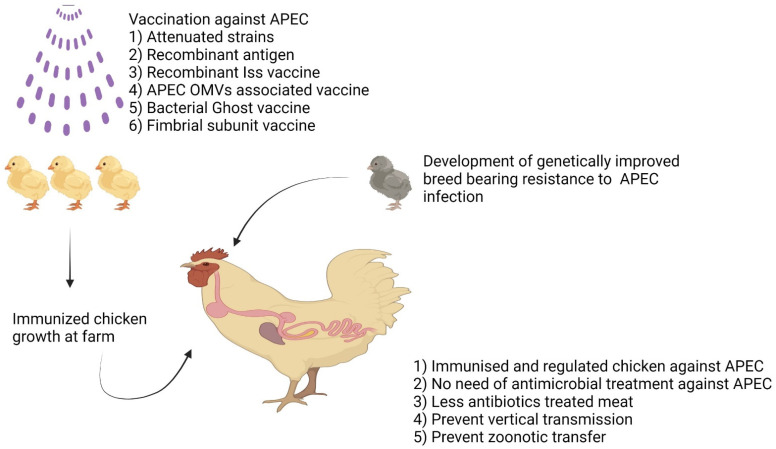
Vaccine development strategies against APEC. Different approaches used for vaccination against E. coli in poultry include live-attenuated vaccines (attenuation by chemical mutagenesis, or specific genetic modifications causing decreased virulence), inactivated or killed vaccines (whole bacterial cells killed by heat, chemicals such as formaldehyde, acetone, alcohol, irradiation, etc.), toxoids (toxins rendered innocuous by heating or formalin), or subunit-based vaccines (prepared from immunogenic epitopes of whole-cell proteins, outer membrane proteins, flagellin, fimbriae, lipopolysaccharides), and recombinant DNA technology (DNA vaccines, mRNA vaccines). These vaccines are normally administered by spray using coarse aerosol sprayer machine or mixed in drinking water free of chlorine or other sanitizing agents to one-day-old chickens or within the first week after hatching. The figure was created with BioRender.com (accessed on 22 January 2023).

After registration in the EU in 2013, O78 (Poulvac^®^ *E. coli*, Zoetis) is commercially used as a vaccine, which is a live-attenuated *aroA*-mutant of an *Escherichia coli* serogroup O78 strain [438]. The vaccine is available as a freeze-dried powder (lyophilisate) and is given as a single-dose vaccine either by spray application with chicks from one day of age or by adding to the drinking water of chicks from five days of age. One important limitation of this vaccine that has been reported is limited protection against the diversity of APEC belonging to a multitude of different serotypes [439,440]. There is, therefore, a need to develop a vaccine against APEC that would provide broader spectrum protection against multiple serotypes. Another vaccination strategy is the use of autogenous killed bacterial isolates as vaccines, which can provide an important means of reducing recurring APEC infections in poultry rearing and production facilities [441]. Autogenous vaccines have a long tradition, and the use of killed pathogenic strains as vaccines isolated from the farm of origin can provide a decrease in recurring outbreaks. Both risks and benefits are associated with this technique, with the major risk being the potential transmission of viral, bacterial, and/or fungal contaminants [442]. On the positive side, autogenous vaccination can provide an important protective advantage when there is a sudden outbreak, and other commercially available vaccines could not adequately protect due to the heterogeneity of the APEC outbreak strains. Other techniques include the use of inactivated or killed vaccines prepared from the whole bacterial preparation combined with adjuvant and subunit vaccines prepared from outer membrane proteins, whole-cell proteins, flagellin, pilus proteins, or LPS [443,444] (Figure 5). It is important to note that although vaccination may provide some level of potential protection against colibacillosis, proper management and biosecurity in the poultry farm are paramount for maintaining good poultry health. Some of the ongoing research regarding vaccines and vaccine development against APEC is presented in Table 6.

### 4.2. Porcine Colibacillosis

Porcine colibacillosis are enteric infectious diseases caused by pathogenic *E. coli* which accounts for one of the major diseases affecting the swine industry. Colibacillosis in swine includes mainly neonatal diarrhea and post-weaning diarrhea, resulting in illness and death occurring worldwide in neonatal and recently weaned pigs. Neonatal swine diarrhea is observed in piglets aged 1–4 days after farrowing, pre-weaning diarrhea within 1–2 weeks post-farrowing to weaning, post-weaning diarrhea occurs 2–3 weeks after weaning with a peak rise of diarrhea from 6 to 8 weeks and even at 12 weeks after weaning [452,453,454]. In addition, Edema disease is usually observed after weaning and other *E. coli* infections such as mastitis, UTIs (cystitis) and septicemia are also observed. Although EHEC, EAEC and EIEC are also known to cause pathogenesis in pigs; ETEC and EPEC are major pathovars that cause serious health problems and even death in piglets around the world. Enterotoxigenic *E. coli* (ETEC) is the most important pathotype and is commonly associated with post-weaning diarrhea (PWD) and neonatal diarrhea. Cases of PWD have also been associated with EPEC [455,456,457]. Neonatal diarrhea and PWD are the most prevalent porcine diseases, accounting for substantial economic losses worldwide [458]. Common serotypes of ETEC causing neonatal and post-weaning diarrhea include O8: K88, O149: K88 and O157: K88 [455,456,459]. In addition, many other serotypes are associated with ETEC infections in piglets. Apart from ETEC and EPEC, Verotoxigenic *E. coli* is also known to be associated with PWD, edema disease and other related infections in swine. Among verotoxigenic *E. coli* isolates of weaned pigs with enteric diseases, common serotypes include O157: K17, O149:K91 and O groups 138, 139 and 141 [460,461,462,463].

Adhesins and enterotoxins are major virulence factors of *E. coli* causing diarrheal disease in pigs. Adhesins contribute to the colonization, proliferation and development of infection, and enterotoxins also play a role in the colonization and development of disease [456]. ETEC strains can produce a number of different fimbrial adhesins. Fimbriae in ETEC are divided into four main types: F4 (K88), F5 (K99), F6 (987P), and F41. The ETEC strains also produce LT and ST (STa and STb) enterotoxins. It has been reported that ETEC strains producing F4 fimbrial adhesins and producing LT and ST enterotoxins are responsible for causing diarrhea in neonatal, pre- and post-weaning pigs [464,465]. In addition, EPEC strains with F41 and F18 fimbriae and producing STb enterotoxin STb are known to cause swine diarrhea. Moreover, VTEC strains with F4 (K91) fimbriae and LT and ST enterotoxins are also reported to cause infections in pigs [463,466,467].

#### 4.2.1. Resistance to Multiple Antibiotics in *E. coli* Causing Diarrhea in Swine

Swine production is one of the major sources of animal meat production. Over the years the presence of bacterial diseases has resulted in increased use of antibiotics to prevent such infections and reduce economic losses and improve animal health. The wide use of antibiotics has consequences, and there is a global concern in increasing antibiotic resistance. It has been reported that there is a high level of antibiotic resistance in porcine *E. coli* isolates from China [468,469,470,471,472,473]. Antibiotic resistance in *E. coli* is also prevalent on pork farms across Europe, including Spain [455,474], and other pork exporting countries such as Denmark, France, Italy, and Sweden are also found to be multi-drug resistant to antibiotics such as ampicillin, streptomycin, sulphonamide, tetracycline, and trimethoprim [475].

#### 4.2.2. Need of Vaccines and Vaccine Strategies against Porcine Pathogenic *E. coli*

The pork industry is one of the most important meat industries in terms of numbers and biomass. The UN Food and Agriculture Organization has predicted that the pork meat industry will experience strong growth among all meat industries with an expected increase of 8.6% by 2030 and 12.7% by 2050. It is therefore crucial to understand and structure sustainable growth of the pork production industry in terms of animal health, antibiotic resistance, and alternative routes to the antibiotic through the development and implementation of vaccines and other approaches to improve porcine health and provide a high-quality consumer product globally.

Since adhesins and enterotoxins are vital for ETEC pathogenesis and virulence, various approaches have been developed to inhibit the initial stages of ETEC pathogenesis by developing anti-adhesin and anti-toxin-based vaccines [476,477]. Over the past decade, multiple vaccination strategies have been developed. For example, a live-attenuated *S. typhimurium* Δ*lon* Δ*cpxR* Δ*asd* strain has been used to deliver ETEC fimbriae in order to promote an anti-adhesin-based vaccine immune response in swine. This vaccine showed no clinical signs of diarrhea in the vaccinated and challenged group and generated increased levels of IgG and IgA in both serum and colostrum [478]. Some porcine ETEC strains produce enterotoxins but use fewer common types of fimbriae, so it is important to design a vaccine that can provide a large spectrum of protection against multiple types of porcine pathogenic *E. coli*. One such example is a live-attenuated *E. coli* strain expressing an adhesin-toxoid fusion antigen that has been shown to elicit a good systemic and mucosal antibody response and protect piglets from ETEC-associated diarrhea [479]. Another group designed a multivalent vaccine candidate comprising STa-LTB-STb (SLS) toxin-based antigens, and two fimbrial proteins, which showed significant protection against an ETEC strain [480]. Likewise, a peptide-based vaccine constructed by genetically fusing nucleotides encoding peptides for fimbria and toxins to obtain tripartite adhesion-adhesin-toxoid chimeric antigens were given to piglets and they provided protection against a challenge using a fimbrial/LT/STb producing ETEC strain [481]. This study demonstrated that multiple adhesin antigens and multiple toxin antigens could be expressed simultaneously within a single recombinant fusion protein. In the future, non-pathogenic *E. coli* isolates from a farm that expresses a tripartite antigen could also be used to develop a live attenuated vaccine against porcine ETEC. Along with these, other strategies such as an oral fimbrial subunit vaccine, live oral vaccine, encapsulated subunit, and other live vaccine candidates are described in detail in the following reviews [453,459,476,482].

Another interesting strategy that is being considered is a parenteral vaccine which is based on the presence of lactogenic maternal antibodies via parenteral immunization. The entero-mammary route allows oral maternal immunization to elicit a mucosal and systemic immune response to reach the gut of the suckling piglet with humoral and cellular components with effector activity [482]. This will provide protection against infection by either maternal antibodies and leukocytes present in the suckling infants or by acquiring specific piglet immunity through the sow. Research has suggested that suckling piglets born from immunized sows with Sta toxoid fusion antigens were protected from a challenge with an STa-positive ETEC strain [483]. Another vaccine candidate containing a Sta toxoid fusion and chemical conjugates was used to immunize sows, and the suckling piglets passively acquired anti-STa IgG and IgA antibodies and were better protected from challenges with an STa positive ETEC strain [484,485]. Some popular commercially available vaccines are manufactured by Elanco and Vencofarma and are composed of inactivated ETEC bacterins which are administered to pregnant pigs two weeks before farrowing.

Overall, some approaches to vaccination against porcine *E. coli* have shown promise against some common types of strains causing swine diarrhea. However, due to the diversity of pathogenic *E. coli,* certain strains are still a problem to the industry. Importantly, a global and integrative approach is needed to regulate and monitor multiple aspects from sanitation, feed regimen, antibiotic usage, and vaccine strategies to maintain and improve the production of sustainable pork production industry worldwide.

### 4.3. Bovine Colibacillosis

Bovine colibacillosis is an infection caused by ETEC strains. Although EPEC, EHEC and VTEC strains are commonly present in the bovine intestinal tract [486], they are generally considered non-pathogenic to cattle [487]. Enteric colibacillosis is usually observed in young calves that are from 2 to 10 days old [488]. Stress can also lead to ETEC-associated diarrhea in older calves. During the infection, calves suffer from profuse and watery diarrhea, become dehydrated, depressed, anorexic, do not suckle and may die rapidly. Depending on the severity of symptoms, calves frequently die in 3 to 5 days [489]. Colibacillosis outbreaks in cattle have been associated with multi-resistant strains under poor sanitation conditions and with susceptible calves in the herd population [490,491]. It has been shown that calves of first-calf heifers are more susceptible to infection, mainly due to the presence of lower levels of immunoglobulin in colostrum and the production of less colostrum itself [492,493]. Calves are also known to develop septicemic colibacillosis due to invasive serotypes of ETEC that can enter the bloodstream and cause systemic infection and rapid death due to septicemia [494,495]. Generally, ETEC strains adhere to the ileum of calves and release toxins to induce severe diarrhea. Septicemic *E. coli* strains can cause systemic infection and, in some cases, EHEC and VTEC strains produce Shiga-toxins that destroy gut microvilli and can cause hemorrhagic diarrhea in 15-to-30-day-old calves [496,497].

Bovine ETEC isolates from diseased calves commonly produce K99 (F5) adhesin and heat stable (STa or STb) or heat liable (LT1 or LT2) enterotoxins. It has been reported that out of 173 isolates of ETEC 49% of strains produced toxins where 53 isolates harbored Sta toxin and 9 contained both STa and LT toxin encoding sequences. Interestingly, nine isolates harbored Shiga-toxin genes [491,498,499,500,501,502]. There is also a high incidence of multiple drug resistance in bovine ETEC isolates throughout the world [490,503,504,505,506,507,508,509]. With an increased concern due to multiple antibiotic resistance, there is a greater interest in vaccines and other preventative measures to reduce the incidence of colibacillosis in calves (See Table 6 which lists such vaccine strategies).

### 4.4. Mastitis in Cattle and Swine

Mastitis is a disease characterized by intramammary infections caused mainly by microorganisms and, in some cases, worsened by physical trauma that results in persistent inflammation of the udder and mammary tissue [510]. Microorganisms such as viruses, fungi, mycoplasma, and numerous bacterial species can cause mastitis. *E. coli* are a common cause of mastitis [170,511,512]. Mastitis in swine is one of the causes of maternal and pre-weaning piglet mortality [513]. *E. coli* is known to be one of the leading causes of inflammatory infection of mammary glands in sows, resulting in abnormal and decreased milk production and even death in severe cases [514]. Among different pathotypes of *E. coli*, a novel MPEC (Mammary pathogenic *E. coli*) is one of the most common causative agents of coliform mastitis [514]. MPEC strains have evolved by acquiring virulence factors that promote colonization and invasion of the mammary glands and increase survival in milk [515,516,517].

Due to excessive use of antibiotics to treat bovine mastitis, there has been an increased concern about the presence of antibiotic-resistant bacteria on dairy farms and in milk and dairy products [518,519,520,521]. Therefore, to overcome this challenge, different vaccination approaches to prevent coliform mastitis have also been tested. Commercially, mutant strains of *E. coli* O111:B4 (J5) [522] and *Salmonella* Typhimurium Re-17 [523] are being used to immunize cows against coliform mastitis. In addition, recombinant OmpA (outer membrane protein A) is also being studied as a potential vaccine candidate to prevent infection against mastitis-associated *E. coli* [512,524]. Other vaccination strategies against mastitis caused by organisms other than *E. coli* are also described in Table 7.

## 5. Conclusions

The diversity of pathogenic *E. coli* which collectively includes distinct pathotypes of importance for both human and animal health has been and remains an important challenge for the development of effective vaccines. Identifying protective and conserved antigens to prevent specific types of *E. coli* disease is complicated by the different types of diseases as well as the heterogeneity of gene content contributing to the pathogenic potential among, and even within, pathotypes associated with distinct disease syndromes in either humans or other animals. Thus far, for vaccine candidates to prevent human infections, almost all the studies have been in animals and the few human studies conducted to date have either been failures, generated inconclusive data, or have not been further pursued. Based on the aforementioned diverse nature of *E. coli* virulence factors, one can predict that an “ideal” vaccine that can prevent multiple types of *Escherichia coli* infection and disease is unlikely. However, the development of different vaccine types and innovative immunization strategies appears promising. A major hurdle in overcoming *E. coli* pathotypes in some cases is the presence of multiple serotypes that are responsible for either human or animal infections. Surface-exposed components are often attractive and effective candidates or vaccine development, but in the case of *E. coli* vaccines, particularly in humans, the selection of targets more specific to pathogenic strains may be a more favorable approach, than targeting highly conserved antigens, considering that *E. coli* strains are also common members of the intestinal microbiota. As such, the generation of an immune response to highly conserved antigens in most *E. coli* and other enterobacteria may be protective against some types of infection but may also affect the host intestinal mucosal immune response and colonization of commensal *E. coli* in the intestinal tract. Such aspects of the effects of selection of highly conserved vs. more patho-specific antigens need to be more fully investigated in both animal models and humans in future studies. In addition to the vaccine development strategies presented herein, it will be of interest as well to investigate how other novel vaccine approaches such as mRNA vaccination may provide protection against certain pathotypes of *E. coli*. However, the use of such vaccines may be limited due to cost or issues with vaccine stability and the requirement of a cold chain particularly for vaccination of populations in outlying regions and in developing countries where endemic infections such as diarrhea caused by intestinal pathogenic *E. coli* and other pathogens remain a global health challenge. Apart from vaccination campaigns, different preventive strategies such as improved sanitation and hygiene, access to safe drinking water, exclusive breastfeeding, optimal nutrition, and vaccines against other pathogens (rotavirus and measles) are equally important. In any case, continued development of new strategies for *E. coli* vaccine discovery is warranted in the context of its antibiotic resistance. Hopefully such strategies will lead to the development of more effective means to reduce the incidence of the spectrum of infectious diseases that *E. coli* can cause in both humans and animals of agricultural importance which remain a globally important economic and public health burden.

## Figures and Tables

**Table 1:** Reports describing vaccines against Enteropathogenic *E. coli* (EPEC).

Type of Vaccine	Component of Vaccine	Results/Observations/Outcomes	Animal Model (Year)	References
Antigen-Based Vaccine	Recombinant *Mycobacterium smegmatis* (Smeg) and *Mycobacterium bovis* BCG to express BfpA or intimin.	Yielded high titer of IgG and IgA antibodies in the serum of immunized mice. Mice immunized with recombinant BfpA showed TNF-α and INF-γ, and TNF-α only with recombinant intimin.	Mice immunized by oral gavage or intraperitoneal injection (2012).	[91]
	Combination of purified recombinant EspA, Intimin, and Tir.	Showed protection of immunized cattle against O157 challenges.	Male Holstein-Friesian calves immunized orally (2010).	[97]
	*Lactobacillus casei* expressing intimin-β and immune-dominant isotopes of Int280.	Induced cellular and humoral responses in mice. Serum antibodies inhibited EPEC adhesion to epithelial cells in vitro.	Mice immunized intranasally (2008).	[98]
Plant-based vaccine	Transgenic plants expressing intimin, and BfpA.	Proposed edible vaccines under strategic and regulation planning.	Mice immunized orally (2002).	[99]
Live-attenuated bacterial vaccine	Live attenuated Δ*espF*Δ*ushA Citrobacter rodentium* strain.	Oral administration in mice yielded efficient systemic and humoral immunity against *C. rodentium* virulence factors.	Mice immunized by oral gavage (2022).	[100]
Adjuvanted whole-cell vaccine	Cholera toxoid (CTB)-adjuvanted formalin-killed whole bacterial cell (EPEC).	100% survival rate of Balb/C mice when challenged with EPEC.	Mice immunized intraperitoneally (2016).	[101]

**Table 3:** Reports describing vaccines against Enterotoxigenic *E. coli* (ETEC).

Type of Vaccine	Component of Vaccine	Results/Observations/Outcomes	Animal Model/Phase (Year)	References
Attenuated bacteria-based vaccines	Attenuated ETEC E1392/75-2A Δ*aroC*Δ*ompR* and ETEC E1392/75-*2A*Δ*aroC*Δ*ompR* Δ*ompC* mutations	Significant yield in IgA and IgG and CS1 and CS3 specific antibodies.	Mice immunized intranasally (2001)	[179]
	ETVAX (attenuated bacteria expressing CS6 in *E. coli* K-12 and CFA/I, CS3, CS5 in ETEC O78 toxin-negative) with LCTBA hybrid protein and mutated Heat-label (LT)	Currently in clinical trials (NCT02531802). High titers of fecal, jejunal and serum IgA and IgG in orally immunized humans.	Phase II clinical trials (2020)	[186,187]
	ACE527 ETEC complex (ACAM2022 (O141:H5, expressing CS5 and CS6), ACAM2025 (O39:H12, expressing CFA/I) and ACAM2027 (O71:H-, expressing CS2, CS3, and CS1)	33–98% protection in reducing the duration of diarrhea in human clinical trials (NCT00901654).	Phase I clinical trials (2015)	[178,188]
	Attenuated ETEC strains expressing CFA/I, CS2 and CS3 and CS1, CS2, and CS3 generating ACAM2010, 2007, 2017 strains respectively	Elevated levels of IgA against CFA in orally immunized human volunteers.	Human volunteers, double-blind trials (2008)	[178]
	Attenuated ETEC strains expressing CS5, CS6, LT, ST and EAST1 generating ACAM2025, 2022, 2027 strains.	Double-blind placebo-controlled Phase II challenge trial.	Phase II clinical trials (2019) NCT01739231	[189,190]
	Strains of *Vibrio cholerae* expressing CFA/I	Significantly yielded high levels of IgA and IgG titers in the serum of immunized mice.	Mice immunized orally (2008)	[191]
	Non-toxigenic *E. coli* expressing CS2, CS4, CS5, or CS6 and CFA/I	Significantly induced IgG+IgM and IgA antibodies CS6 in sera and feces, respectively, in immunized mice.	Oral immunization of mice (2010)	[192,193,194]
Adhesin-based vaccines	Recombinant ETEC expressing two-partner secretion protein A (EtpA)	Less bacterial (ETEC) colonization in the gut of immunized mice.	Intranasal immunization of mice model (2009-2016)	[195]
	CS21/LngA formulated with cholera toxin	Increased specific IgG and IgA in serum and feces and intestinal lavages, respectively. Reduced shedding in immunized mice.	Intranasal immunization of mice (2017)	[196]
OMV-based vaccines	ETEC OMVs Δ*msbB*Δ*eltA*	Detoxified OMVs induced higher titers of IgG1, IgM, and IgA and significantly reduced wild-type ETEC colonization in immunized mice.	Mice immunized intranasally (2015)	[196]
	*Vibrio cholerae* OMVs Δ*msbB*Δ*ctxAB*Δ*flaA* expressing ETEC FliC and CFA/I	OMVs yielded higher titers of IgG1, IgM, and IgA and reduced wild-type ETEC colonization and spread in immunized mice.	Mice immunized intranasally (2015)	[197]
Autotransporter-based vaccines	Recombinant Ag43 and pAT (autotransporters)	Significant increase in fecal IgA and partial protection against intestinal colonization of ETEC in immunized mice.	Mice immunized intranasally (2011)	[198]
Toxin-based vaccines	Heat-labile (LT) toxin using skin patches.	Yielded significant levels of anti-LT IgG and IgA in 97–100% of human volunteers. Currently, in Phase 2, the clinical trial complete phase (NCT00565461).	Phase II clinical trial (2020)	[199]
	STaP13F-LTR192G toxoid fusion protein	Induced IgG-specific antibodies for LT and STa in serum and feces and IgA in feces in immunized mice.	Oral immunization of mice (2019)	[179]

**Table 4:** Reports describing vaccines against Enteroinvasive *E. coli* (EIEC)/*Shigella*.

Type of Vaccine	Component of Vaccine	Results/Observations/Outcomes/Animal Model/Phase (Year)	References
Live-attenuated vaccine	ShigETEC attenuated *Shigella* strain expressing ETEC antigens (LTB and detoxified version of ST)	Currently, phase I clinical trials yielded high titer IgG and IgA against bacterial lysates and anti-ETEC toxins (2022)	[185,232]
	WRSs2 attenuated Δ*virG S. sonnei* in which enterotoxin genes senA/senB are deleted.	Currently, phase II clinical trials, NCT04242264 (2021)	[233]
	WRSs3 *attenuated* Δ*virG S. sonnei* in which *senA/senB*, and acetyl transferase genes *msbB* are deleted.	Currently, phase II clinical trials (2021)	[233]
	*Shigella flexneri* 2a, O antigen mutant (Δ*wzy*) combined with *E. coli* LT mutant.	Provided cross-protective immunity against Shigella and ETEC. Pre-clinical stage (2018)	[234]
	The formalin-inactivated trivalent *Shigella* whole-cell vaccine	Phase II clinical trials (2022)	[224]
Heat-killed multi serotype	Heat-killed cocktail of 6 strains of *Shigella* inactivated vaccine	Pre-clinical stage (2016)	[223]
Subunit vaccines	S4V-EPA, four-valent, O-antigen bioconjugates against *S. sonnei*, *flexneri 3a* and *flexneri 6*, and *S. flexneri 2a*.	Phase II clinical trials (2022)	[235] Limmatech
	SF2a-TT15, *S. flexneri 2a* synthetic O-antigen conjugates against *S. flexneri 2a*	Phase II clinical trials (2022)	[236]
	InvaplexAR-DETOX, artificially detoxified *S. flexneri 2a* invasin complex with recombinant IpaB/IpaC	Phase I clinical trial (2021)	WRAIR, [233]
	ZF0901, Bivalent O-antigen glycoconjugate against *S. flexneri 2a* and *S. sonnei*	Phase III clinical trial (2021)	Beijing Zhifei Lvzhu biopharmaceuticals, [237]
	altSonflex 1-2-3, a four valent Shigella native outer membrane vesicle (OMVs) against *S. flexneri 1b, 2a, 3a* and *S. sonnei*.	Phase II clinical trial (2022)	[224]
Adjuvanted whole-cell vaccine	Alum-adjuvanted and CTB-adjuvanted EIEC whole-cell vaccine	Higher IgG yield and immune response against EIEC and ETEC in orally immunized mice (2016)	[101]

**Table 5:** Reports on vaccines and other strategies against Adherent invasive *E. coli* (AIEC) and Inflammatory bowel disease (IBD).

Type of Vaccine	Component of Vaccine	Results/Observations/Outcomes	Animal Model (Year)	References
Adjuvanted enhanced vaccine	Intranasal immunization of mice using siderophore enterotoxin (Ent) conjugated with CTB.	Increased fecal antibodies against Ent and reduced AIEC colonization in immunized mice.	Orally immunized mice (2021)	[285]
	CTB-Ent, immunization of mice.	Mucosal IgA against Ent and GlcEnt, protection from systemic infection and decreased AIEC and Crohn’s disease and colitis in mice.	Orally immunized mice (2022)	[286]
Inhibition of FimH adhesin	Thiazolylaminomannosides and n-heptyl α-D-mannose based inhibition of AIEC LF82 adherence to colon tissue by blocking FimH	FimH blocker molecule EB8018/TAK-018 is under phase 2a clinical trial (NCT03943446)	Clinical trial (2020)	[262]
Probiotics, prebiotics, and postbiotics	Probiotics containing a portion of *S. cerevisiae* CNCM I-3856	Known to prevent colitis induced by AIEC in the mouse model of Crohn’s disease.	Orally immunized mice (2018)	[287]
	Probiotics *Lactobacillus rhamnosus* GG and *Lactobacillus reuteri.*	Known to reduce AIEC survival and growth.	Orally immunized mice (2018)	[288]
	Prebiotics containing long-chain arabinoxylans	Known to inhibit the mucin adhesion of AIEC.	Orally immunized mice (2020)	[289]
	Prebiotics containing insulin and galacto-oligosaccharides	Limit AIEC survival and growth	Orally immunized mice (2017)	[290]
	Postbiotics such as colicins E1 and E9 that are species-specific bacteriocins.	Known to kill intracellular, biofilm-forming and cell-adhering AIEC.	Mice model (2018)	[291]
Fecal microbiota transplantation (FMT)	Restoration of normal intestinal flora to prevent CD and AIEC colonization	Placebo-controlled trials using FMT have shown improvements in patients with active disease.	Pre-clinical trial stage (2022)	[292]
Phage Therapy	LF82 bacteriophages that were able to replicate in ileal, colon samples and feces.	An oral dose of bacteriophages has inhibited AIEC strain LF82 colonization and colitis symptoms in the gut. Phase 1/2a clinical trial is ongoing.	Human volunteers (2015)	[293] Mount Sinai hospital
	Five-phage cocktail against IBD	IBD suppression. Currently in Phase II clinical trial (NCT04737876).	Clinical trial (2022)	[294]

**Table 6:** Avian colibacillosis treatment/vaccine strategies.

Type of Vaccine	Component of Vaccine	Results/Observations/Outcomes	Animal Model (Year)	References
Bacterial ghost of APEC+A106:D112	Design of bacterial ghost of *E. coli* O78:K80 by making the porous cell wall	Aerosol-vaccinated chickens challenged with APEC O78:K80 had reduced air sac lesions and less death. Vaccinated chickens showed increased levels of IFNγ, IgA and IgY.	Broiler chicken (2018)	[445]
	Nucleic acid-free bacterial ghost vaccine of *E. coli* O78:K80 by removing cytoplasmic content and nucleic acids.	Chickens vaccinated by injection or inhalation both showed humoral and cellular immune responses and cytokine responses. Challenge with O78:K80 showed lower lesion scores and bacterial numbers in the vaccinated group.	Broiler chicken (2020)	[446]
Liposomal inactivated APEC vaccine	Liposomal inactivated avian pathogenic *Escherichia coli* (APEC) strain containing vaccine.	Chickens vaccinated via eye drops or coarse spray produced anti-LPS antibodies (IgG) in serum and IgA in oral mucus.	Broiler chicken (2009)	[447]
Recombinant attenuated Salmonella vaccine (RASV)	RASV-producing *E. coli* common pilus (ECP) and booster dose with a combination of RASV χ8025(pYA3337) and χ8025(pYA4428) or χ8025(pYA3337), RASV χ8025(pYA4428) carrying *ecp* operon genes.	Chickens were vaccinated orally and challenged with APEC O2 or O78 strain via air sac. Immunized chickens after vaccination showed significantly increased levels of serum (IgY) and intestinal (IgA) antibody. Challenged chicken showed partial protection against APEC.	White Leghorns chicken (2018)	[448]
	RASV with Δ*lon,* Δ*cpxR,* and Δ*asdA16* and producing P-fimbriae, aerobactin receptor, and CS31A surface antigen of APEC.	Immunized chickens showed increased IgG and IgA antibodies. Chickens were challenged via the air-sac route and showed partial protection against virulent APEC.	Broiler chicken (2013)	[449]
	RASV plus commercial probiotics supplements	White Leghorn Chickens are given RASV, and probiotics elicited significant serum and mucosal antibodies. When challenged with APEC virulent strains showed lower bacterial loads and lesions of airsacculitis and pericarditis/perihepatitis.	White leghorn chicken (2020)	[450]
Outer membrane vesicle (OMV) based vaccine	Purified OMVs from O1, O2, and O78 strains to develop a multi-serogroup vaccine (MOMVs).	Vaccinated chickens effectively yielded specific antibody responses against each OMV antigen. They also yielded significant cellular and humoral immune responses. Immunization with MOMVs showed 100%, 90% and 100% cross-protection against challenge by O1, O2 and O78 APEC strains.	Broiler chicken (2020)	[451]

**Table 7:** Reports concerning bovine colibacillosis treatment/vaccine strategies.

Type of Vaccine	Component of Vaccine	Results/Observations/Outcomes/Year	References
ScourGuard^®^ 4K	Cocktail of inactivated bovine rotavirus, coronavirus and *E. coli* bacterin	Vaccination of healthy pregnant cows prevented diarrheal disease in calves bovine ETEC bearing K99 pili, bovine rotavirus (serotypes G6 and G10), and coronavirus (2021)	[525] (Zoetis, USA)
ScourGuard^®^ 4Kc	Cocktail of inactivated bovine rotavirus, coronavirus, *Clostridium perfringens* type C and *E. coli* bacterin-toxoid	Vaccination of healthy pregnant cows prevented diarrhea caused by bovine rotavirus (serotypes G6 and G10), coronavirus, ETEC and *C. perfringens* in calves given colostrum from vaccinated mother (2021)	[525] (Zoetis, USA)
Bolus and Dual-force gel	Contains passive antibodies against diarrheal pathogens	A single dose administered after birth protects calves from *E. coli* and coronavirus infection (2021)	First Defense
Tri-Shield First defense	Contains passive antibodies against diarrheal pathogens. Should be administered with maternal colostrum	A single dose administered after birth provides passive immunity against K99+ *E. coli*, coronavirus and rotavirus (2021)	First Defense
First Defense Technology	Hyper-immunized colostrum antibodies	Antibodies that neutralize *E. coli* and coronavirus and provide instant immunity (2021)	First Defense
Bioniche^®^ vaccine	A Type III secretion system-based vaccine	Reduces *E. coli* O157:H7 (EHEC) growth and colonization in cattle (2013)	[526] Bioniche
Epitopix^®^ vaccine	Siderophore receptor and porin protein (SRP) vaccine	Reduces *E. coli* O157:H7 (EHEC) growth and colonization in cattle (2018)	[527]
Fencovis^®^ vaccine	Administered to pregnant cows to provide passive immunity to newborn calves via maternal colostrum	Active immunization of cows stimulates the development of antibodies against *E. coli* F5, rotavirus and coronavirus and prevents neonatal diarrhea (2022)	[528]
J-VAC^®^ vaccine	Broad spectrum adjuvanted bacterin-toxoid	Prevents bovine mastitis caused by *E. coli* and endotoxemia caused by *E. coli* and *Salmonella Typhimurium* (2022)	[529]
Bar-Guard-99™ vaccine	Utilizes whole cell antibodies	Provide rapid passive immunity against *E. coli* K99 and diarrheal diseases (2022)	[530]
ENVIRACOR^®^ J-5 vaccine	Bacterin-based vaccine	Controls clinical signs related to bovine mastitis caused by *E. coli* (2021)	Zoetis, USA
BOVILIS^®^ J-5 vaccine	Endotoxin-based vaccine	Prevents milk loss, culling and death related to bovine mastitis caused by *E. coli* (2021)	MERK animal health
DNA and subunit-based vaccine	Lipopolysaccharide-based pcwaaF (DNA vaccine) and rwaaF (recombinant waaF subunit vaccine)	Greater IgG, IL-2, IL-4, and IFN-γ, and fecal sIgA. Mice survive better post-challenge with mastitis, causing *E. coli* (2022)	[531]
Proteo-liposome based vaccine	Proteo-liposome extracted from bovine mastitis clinical isolate (RM5870)	A significant level of IgG, IgG1 and IgG2a, IgA. Improved survival rates of mice post-challenge with *E. coli* causing mastitis. Reduced bacterial loads, inflammation, and tissue damage in mammary glands (2022)	[532]

## Data Availability

Not applicable.
